# Microbiome Diversity in Pancreatic Surgery: Associations with Preoperative Stenting and Postoperative Outcomes

**DOI:** 10.3390/microorganisms14050951

**Published:** 2026-04-23

**Authors:** Laura Oelschlägel, Johannes Klose, Markus Glaß, Stefan Moritz, Bogusz Trojanowicz, Jörg Kleeff, Artur Rebelo

**Affiliations:** 1 Department of Visceral, Vascular and Endocrine Surgery, University Hospital Halle (Saale), Martin-Luther-University Halle-Wittenberg, Ernst-Grube-Str. 40, 06120 Halle (Saale), Germanyjohannes.klose@uk-halle.de (J.K.); stefan.moritz@uk-halle.de (S.M.); bogusz.trojanowicz@uk-halle.de (B.T.); joerg.kleeff@uk-halle.de (J.K.); 2 Institute of Molecular Medicine (IMM), Section for Molecular Cell Biology, Halle-Wittenberg, Charles Tanford Protein Centre, Martin-Luther-University, 06120 Halle (Saale), Germany; markus.glass@medizin.uni-halle.de

**Keywords:** pancreatic surgery, microbiome, bile microbiota, pancreatic fluid microbiota, 16S rRNA sequencing, biliary stenting, postoperative complications, pancreatic cancer, bile duct cancer, *Enterococcus faecalis*

## Abstract

Carcinomas of the pancreas and bile duct remain highly lethal malignancies, with surgical resection representing the only potentially curative treatment. Despite improvements in perioperative mortality, postoperative complications remain frequent and negatively affect long-term outcomes. Recent evidence suggests that the pancreas and bile ducts harbor distinct microbial communities, challenging the traditional concept of sterility in these environments. However, their composition and clinical relevance remain incompletely understood. This study aimed to characterize microbiome profiles across different anatomical sites in patients undergoing pancreatic surgery, evaluate the impact of preoperative biliary stenting, and assess associations between prevalent bacterial species and postoperative outcomes. A total of 224 samples (bile, pancreatic fluid, duodenal tissue, tumor tissue, and healthy pancreatic tissue) from 58 patients with pancreatic cancer, bile duct cancer, chronic pancreatitis, or healthy pancreas were analyzed using 16S rRNA gene sequencing. Microbial diversity was assessed using the Shannon index for alpha diversity and nMDS with PERMANOVA for beta diversity. Distinct microbial profiles were identified across body sites, with significant beta-diversity differences between duodenal, bile, and pancreatic fluid samples and between duodenal and pancreatic fluid samples from the same patient. Preoperative biliary stenting significantly influenced microbial composition. *Enterococcus faecalis* was associated with a reduced risk of severe postoperative complications (Clavien–Dindo ≥ III). Overall, microbial composition varies across anatomical sites and disease entities, and specific bacteria may influence surgical outcomes, warranting further investigation in larger cohorts.

## 1. Introduction

Pancreatic malignancies are associated with a poor prognosis and remain among the leading causes of cancer-related mortality worldwide. This is largely attributable to late diagnosis, driven by nonspecific clinical symptoms, the absence of reliable early tumor markers, and limitations in imaging modalities for detecting early-stage disease. Consequently, only a minority of patients are eligible for potentially curative surgical treatment at the time of diagnosis [1].

The role of microorganisms in carcinogenesis has been recognized for decades. In 2008, approximately two million of the 12.7 million newly diagnosed cancer cases worldwide were attributed to infections, with nearly 1.9 million linked to pathogens such as hepatitis B virus (HBV), hepatitis C virus (HCV), human papillomavirus (HPV), or *Helicobacter pylori*. To date, eleven so-called oncomicrobes have been identified by the International Association of Cancer Registries, accounting for approximately 13% of global cancer cases [2,3].

Recent research has challenged the long-standing assumption that the pancreas represents a sterile environment. Studies investigating pancreatic ductal adenocarcinoma (PDAC) have demonstrated that the intratumoral microbiome is predominantly composed of Proteobacteria [4]. Geller et al. showed that bacteria isolated from PDAC tissue can mediate resistance to gemcitabine in 14 of 15 bacterial cultures (93%), and bacterial DNA was detected in 76% of tumor samples, with Gammaproteobacteria representing the dominant taxonomic group compared with only 15% in healthy pancreatic tissue [5].

Proteobacteria are also physiologically present in the duodenum, suggesting that retrograde bacterial migration from the gastrointestinal tract may contribute to pancreatic colonization [4,5]. Pushalkar et al. further demonstrated that bacteria can translocate from the gut to the pancreas, with PDAC tissues showing higher bacterial abundance than controls and a microbiome dominated by Proteobacteria (45%), Bacteroidetes (31%), and Firmicutes (22%). In murine models, a stage-specific gut and pancreatic microbiome promoted PDAC progression through intratumoral immune suppression. Conversely, antibiotic-mediated depletion of bacteria enhanced anti-tumor immunity by increasing CD4^+^ and CD8^+^ T-cell infiltration while reducing immunosuppressive myeloid cells [6].

However, emerging evidence suggests that microbial composition may also exert protective effects in pancreatic cancer. Riquelme et al. reported that increased intratumoral microbial alpha diversity was associated with long-term survival in PDAC and identified a potentially beneficial microbial signature including *Pseudoxanthomonas*, *Saccharopolyspora*, and *Streptomyces*. Furthermore, gut microbiota may shape the pancreatic tumor microenvironment, suggesting that microbiome-modulating strategies such as fecal microbiota transplantation (FMT) could represent a future therapeutic approach to enhance immune activation and improve clinical outcomes in PDAC [7].

Cholangiocarcinoma (CCA) represents another highly aggressive malignancy of the hepatobiliary system. Based on anatomical location, CCA is classified into intrahepatic and extrahepatic subtypes, with the latter frequently requiring pancreatic resections because of its proximity to the pancreatic head. Although the biliary tract is physiologically considered sterile, recent studies suggest that dysbiosis and increased intestinal permeability may enable enteric bacteria to migrate into the bile ducts through enterohepatic circulation [8]. Once present in the biliary system, bacterial components such as lipopolysaccharides (LPS) can activate Toll-like receptors (TLRs), triggering chronic inflammation, cholangiocyte proliferation, and potentially malignant transformation [8].

A well-established example is infection with *Opisthorchis viverrini*, a known risk factor for CCA that alters gut microbiota composition, increases *Helicobacter* species abundance, and promotes fibrosis, inflammation, and carcinogenesis [9]. Additionally, increased intestinal permeability facilitates the translocation of LPS into bile ducts, activating TLR4 signaling and recruiting myeloid-derived suppressor cells (MDSCs), which promote angiogenesis, tumor progression, and metastasis. High TLR4 expression has been associated with poor prognosis in CCA patients [8].

Biliary colonization may also occur through iatrogenic mechanisms. Preoperative biliary drainage (PBD) with stent placement is frequently performed to relieve obstructive jaundice in patients with periampullary or pancreatic head tumors. However, this intervention is associated with bacterobilia in nearly 100% of cases compared with approximately 21% in patients without stenting [10]. Stent placement disrupts the natural barrier of the biliary tract and allows retrograde colonization by intestinal bacteria [11]. Furthermore, the presence of foreign material promotes bacterial adhesion and biofilm formation, particularly by species such as *Enterococcus*, which are known to develop antibiotic resistance and create a selective microbial environment [10,12]. Previous studies have also linked PBD to an increased risk of postoperative infectious complications following pancreaticoduodenectomy, although overall mortality appears unaffected [13,14].

In addition to their role in tumorigenesis, microbiome alterations are increasingly investigated as potential biomarkers for cholangiocarcinoma diagnosis and prognosis [15,16,17]. Despite growing evidence suggesting a relevant role of microorganisms in pancreatic and biliary malignancies, the microbial composition across different anatomical compartments and its clinical implications for postoperative outcomes remain incompletely understood.

Therefore, the aim of this study was to characterize the microbial composition of pancreatic, biliary, and duodenal samples obtained during pancreatic surgery and to investigate potential associations between microbial profiles and postoperative complications. In addition, the study evaluates the influence of preoperative biliary interventions, particularly biliary stent placement, on microbial diversity and composition. To address these questions, samples from multiple anatomical sites were analyzed using 16S rRNA gene sequencing.

## 2. Materials and Methods

**Figure 1:**
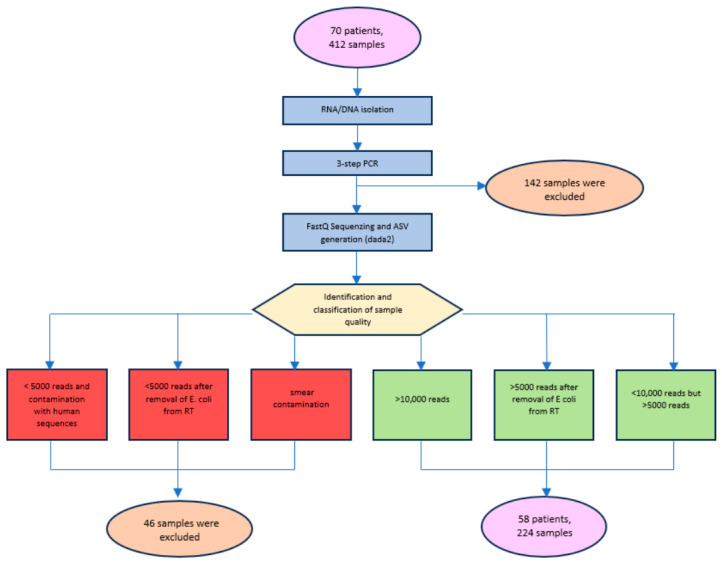
Sample processing and quality control for microbiome sequencing of clinical patient samples. The flow diagram illustrates the processing pipeline from sample preparation to quality control prior to sequence variant analysis within a 16S rRNA sequencing workflow. Red = exclusion criteria; Green = inclusion criteria; Blue = processing steps; Yellow = decision points; Purple/Orange = intermediate results and final inclusion numbers. RT = reverse transcriptase; *E. coli* = *Escherichia coli*.

Between October 2021 and April 2023, 72 patients undergoing pancreatic surgery at University Hospital Halle (Saale) were enrolled (Ethics Approval No. 2019-037). In total 412 samples were collected intraoperatively, including bile fluid, pancreatic secretions, and tissue samples from macroscopically healthy, pathological, and duodenal regions. Samples were analyzed using 16S rRNA gene sequencing, with both DNA and RNA extracted from each sample whenever possible to assess the total and potentially active microbial fractions. Following rigorous quality control, 224 samples were included for taxonomic classification and downstream analyses. A detailed workflow including exclusion criteria is shown in Figure 1. Taxonomic classification was performed based on ASVs using the RDP database. For some bacterial genera, species-level assignment was not possible due to the limited taxonomic resolution of the 16S rRNA gene in the V1–V2 region. This particularly applies to the family Enterobacteriaceae. For example, *Escherichia coli* and *Shigella* cannot be distinguished based on their 16S rRNA sequences and are hereafter referred to as “*Escherichia/Shigella*”. Diversity analyses and logistic regression were performed to assess associations between the microbiome, clinical parameters, and postoperative complications using relative abundances.

Detailed methods are provided in the Methods section of the Supplementary Materials.

## 3. Results

### 3.1. Alpha Diversity Analysis

**Figure 2:**
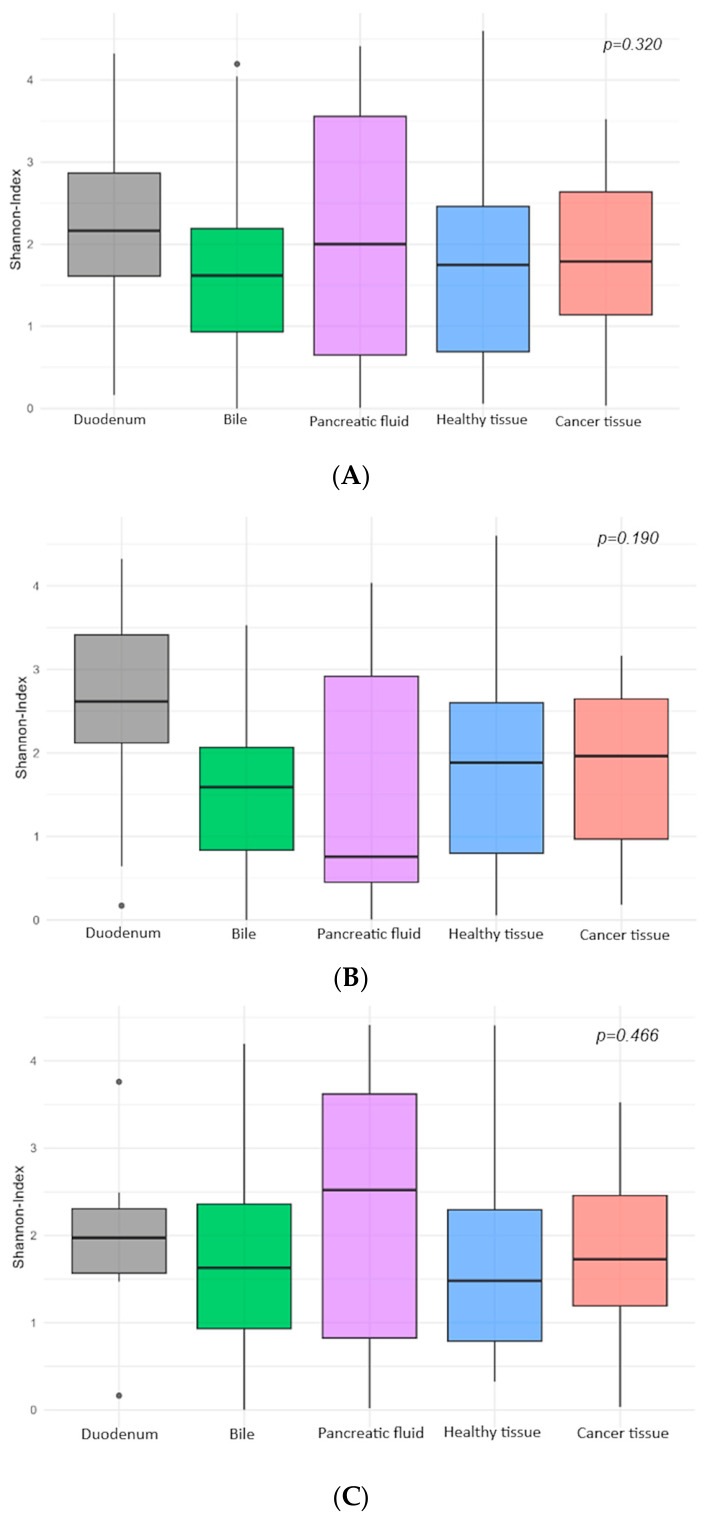
Comparative alpha diversity analysis of different anatomical regions (Shannon Index); (**A**) all samples combined, (**B**) DNA-based samples, (**C**) RNA-based samples.

Alpha-diversity describes the diversity of microorganisms in a single, well-defined habitat. Analyses revealed partially pronounced differences in alpha diversity across the sample groups. Additionally, discrepancies were observed between the results of separate analyses of DNA-based (total microbiome) and RNA-based (active microbiome) samples. Overall, none of the comparisons showed statistically significant differences between the groups (Kruskal–Wallis test, *p* > 0.05). However, descriptive differences between groups were observed (Figure 2).

While healthy and tumorous tissue samples exhibited similar diversity, the most notable differences were observed between pancreatic fluid and duodenal samples. Among DNA-based samples, pancreatic fluid demonstrated the lowest diversity index, yet showed the highest diversity within RNA-based samples. Conversely, the duodenum displayed the highest alpha diversity in the overall and DNA-based analyses but slightly lower diversity in RNA-based samples.

Boxplots further illustrate substantial data variability, with the greatest dispersion observed in pancreatic fluid samples.

### 3.2. Taxonomic Analysis of Defined Groups

In the following, the samples are not evaluated as a whole but are analyzed separately according to anatomical sampling site and diagnosis group. The reported values represent the species-specific mean of all relative abundance values for the respective samples, expressed as percentages.

### 3.3. Taxonomic Analysis by Sample Groups

All bacterial species/genera with a mean relative abundance ≥ 1% were assigned to their respective higher taxonomic levels. Overall, the results demonstrate an individualized microbial distribution of various taxa across different anatomical regions.

**Figure 3:**
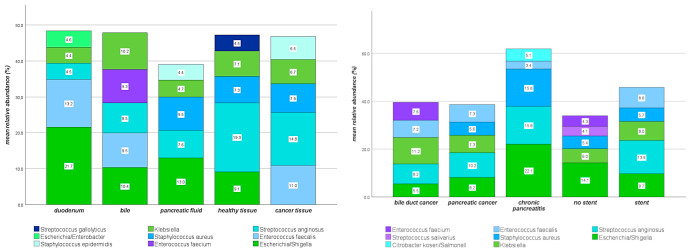
Distinct microbial profiles in different body sites and pancreatic diseases.

In addition, we identified the five most dominant species for each anatomical region based on their average relative abundance (Figure 3). All identified species were consistent with the previous taxonomic results of the overall sample set and were largely dominated by members of the Enterobacteriaceae (e.g., *Escherichia*/*Shigella* and *Klebsiella*) as well as *Enterococcus* and *Streptococcus* species, all belonging to the phyla Proteobacteria and Firmicutes.

Site-specific differences were observed. Duodenal samples were primarily dominated by *Escherichia*/*Shigella* and *Enterococcus faecalis*, whereas biliary communities showed a more even distribution of Enterobacteriaceae and *Enterococcus* species.

In pancreatic fluid, *Escherichia*/*Shigella* remained the most abundant taxon, with additional contributions from *Staphylococcus aureus* and *Streptococcus anginosus*. In both healthy and pathological pancreatic tissue, *Streptococcus anginosus* represented the dominant species.

### 3.4. Analysis Based on Clinical Characteristics

Samples were stratified according to clinical parameters. On the one hand, different pancreatic diseases were analyzed separately; on the other hand, groups were defined based on the presence or absence of preoperative stent placement. The five most dominant species/genera per group were also identified (Figure 3). Samples from patients with histologically confirmed pancreatic malignancy (n = 148 from 39 patients) were primarily dominated by *Streptococcus anginosus*, followed by *Escherichia*/*Shigella*, *Klebsiella*, *Enterococcus faecalis*, and *Staphylococcus aureus*. In patients with cholangiocarcinoma (n = 58 from 11 patients), *Klebsiella* represented the most abundant species, with additional contributions from *Streptococcus anginosus* and *Enterococcus* species (*E. faecium* and *E. faecalis*), while *Escherichia*/*Shigella* occurred at lower abundance. In contrast, samples from patients with chronic pancreatitis (n = 23 from 7 patients) showed markedly higher abundances of *Escherichia*/*Shigella*, *Streptococcus anginosus*, and *Staphylococcus aureus*, each exceeding 10% relative abundance. *Citrobacter koseri*/*Salmonella* and *Enterococcus faecalis* were also present among the dominant taxa. Stratification by stent placement revealed comparable community compositions. In patients with prior stenting (150 samples from 32 patients), *Streptococcus anginosus*, *Escherichia*/*Shigella*, *Enterococcus faecalis*, and *Klebsiella* were the most abundant taxa, whereas samples from patients without prior stenting (74 samples from 26 patients) were primarily dominated by *Escherichia*/*Shigella*, followed by *Klebsiella*, *Staphylococcus aureus*, and *Enterococcus faecium*.

### 3.5. Comparison of Beta Diversity Between Predefined Sample Groups

Beta-diversity measures the species diversity between two or more habitats. A central aim of this study was to compare beta diversity across various predefined sample groups, which were stratified according to different parameters. The interpretation is based on the 2D and 3D non-metric multidimensional scaling (nMDS) plots of all available samples.

### 3.6. Comparison of Beta Diversity Between Duodenal, Pancreatic Fluid, and Bile Samples

#### nMDS Analysis

**Figure 4:**
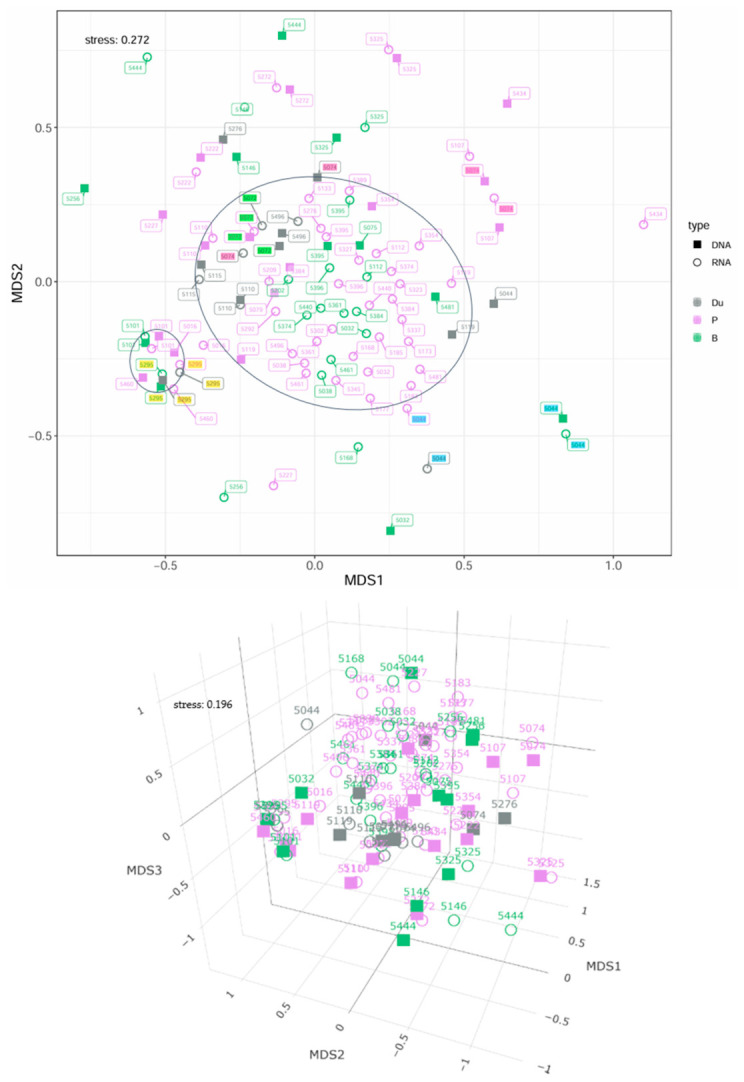
2D and 3D nMDS representation of the microbial composition of duodenal, pancreatic fluid, and bile samples based on Bray–Curtis dissimilarities. Du = duodenum; P = pancreatic fluid; B = bile. Point labels correspond to the respective patient IDs. In the 2D plot, selected patient IDs are color-coded: yellow and blue indicate the two patients with all three sample types available; pink highlights an example of a patient whose different samples are not spatially clustered, while green illustrates an example of a patient whose samples are closely grouped.

Figure 4 presents a 2D and 3D nMDS plot of duodenal, pancreatic fluid, and bile samples from patients without preoperative stent placement. In total, 16 duodenal samples, 59 pancreatic fluid samples, and 30 bile samples from 49 patients were included.

No clear separation between sample types is observed in the 2D nMDS representation; instead, samples formed broadly overlapping groupings. The majority of bile and pancreatic fluid samples were located centrally, particularly RNA-based samples, while DNA-based samples showed greater dispersion. Duodenal samples tended to occur more frequently at the periphery of the central grouping.

No consistent pattern regarding the distance between sample types from the same patient could be established. However, DNA and RNA profiles derived from the same sample generally appeared in close proximity.

The stress value of the 2D nMDS plot was relatively high (0.272), indicating that two-dimensional representation captures the underlying dissimilarities only with limited accuracy. The stress level improved to 0.196 in 3D representation. Nevertheless, bile and pancreatic fluid samples showed substantial overlap at DNA and RNA levels. In contrast, duodenal samples appeared less intermixed, though no clear separation was observed.

PERMANOVA revealed a significant difference in microbial composition between duodenal, bile, and pancreatic fluid samples (*p* = 0.006; F = 1.654), although anatomical grouping explained only a small fraction of variance (R^2^ = 0.031). When analyzed separately, DNA-based samples showed no significant differences (*p* = 0.828), whereas RNA-based samples indicated a trend toward significance (*p* = 0.051).

Including data type and its interaction with group assignment yielded a significant overall effect (*p* = 0.031; R^2^ = 0.060), but neither data type (*p* = 0.522) nor group–data type interactions (*p* = 0.989) were independently significant. In contrast, interindividual differences explained the majority of variance (R^2^ = 0.751; *p* = 0.001).

Intraindividual analyses revealed significant differences between duodenal and pancreatic fluid samples (*p* = 0.003; R^2^ = 0.047), whereas bile and pancreatic fluid samples were more similar, with differences mainly attributable to technical variation rather than sample type.

A comprehensive overview of all PERMANOVA results is provided in the Supplementary Materials.

### 3.7. Comparison of Beta Diversity Between Patients with and Without Preoperative Stent Placement

**Figure 5:**
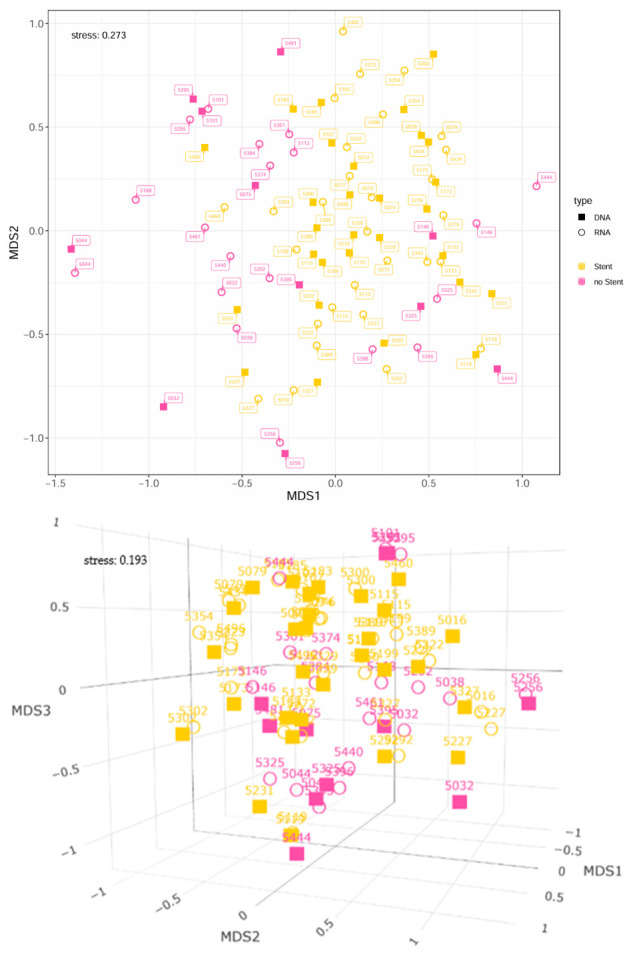
2D and 3D nMDS representation of the microbial composition of patients with and without biliary stents, based on Bray–Curtis dissimilarities using bile samples only. Stent = patients with a history of biliary stent placement; No Stent = patients without biliary stent placement (including patients with a history of pancreatic stenting). Point labels correspond to the respective patient IDs.

Figure 5 compares bile samples from patients with and without biliary stents. A total of 58 samples from 30 patients were assigned to the stent group, and 30 samples from 21 patients to the non-stent group, resulting in 51 patients included in the analysis. In the 2D nMDS plot, a general tendency to group is visible; however, a partial separation between the two patient groups can also be observed. Bile samples from the stent group are primarily distributed within the positive MDS1 region, whereas samples from patients without stents are predominantly located in the negative MDS1 region. Samples from the stent group form a tighter pattern, while those from non-stent patients are more widely dispersed. For each sample, the DNA- and RNA-based analyses are located in close spatial proximity. The stress value for the 2D representation was relatively high (0.273), indicating that the two-dimensional scaling should be interpreted with caution. In the 3D representation, the stress value improved to 0.193, and the separation between the two groupings appeared more pronounced, particularly at the RNA level. In the 3D nMDS plot, stent-group samples are mainly positioned in the positive MDS1 and MDS3 regions, whereas samples from non-stent patients are predominantly located in the negative MDS1 and MDS3 regions. These findings suggest a potential difference in the microbial composition of bile between patients with and without biliary stents, which appears slightly more pronounced at the RNA level.

PERMANOVA revealed a significant difference in microbial composition between patients with and without biliary stents (*p* = 0.001; F = 1.456), although the effect size was small (R^2^ = 0.041). When analyzed separately, significant group differences were observed for both DNA-based (*p* = 0.040; F = 1.702; R^2^ = 0.043) and RNA-based datasets (*p* = 0.001; F = 2.637; R^2^ = 0.054), with stronger effects detected in RNA samples. However, in both analyses, the proportion of explained variance remained low (4.2% and 5.4%, respectively). A multifactorial PERMANOVA including group, data type, and their interaction confirmed a significant overall effect (*p* = 0.001; F = 1.915; R^2^ = 0.064). When tested independently (by = “margin”), the group effect (stent vs. no stent) remained significant (*p* = 0.001; F = 3.561; R^2^ = 0.039), while data type showed no significant influence (*p* = 0.190; F = 1.267; R^2^ = 0.014). Likewise, the group × data type interaction was not significant (*p* = 0.726; F = 0.795; R^2^ = 0.008), suggesting that stent effects were consistent across DNA and RNA datasets. When accounting for interindividual variability by including patient ID in the model, the explained variance increased markedly from R^2^ = 0.064 (6.4%) to R^2^ = 0.889 (88.9%) (*p* = 0.001; F = 5.649), indicating that patient-specific factors dominate the overall variance in bile microbiome profiles.

These findings indicate a statistically significant but modest effect of preoperative stenting on the biliary microbiome, while interindividual differences remain the primary driver of microbial variability. A detailed overview of the PERMANOVA results is provided in the Supplementary Materials.

### 3.8. Comparison of Beta Diversity Between Patients with Pancreatic Cancer and Pancreatitis

**Figure 6:**
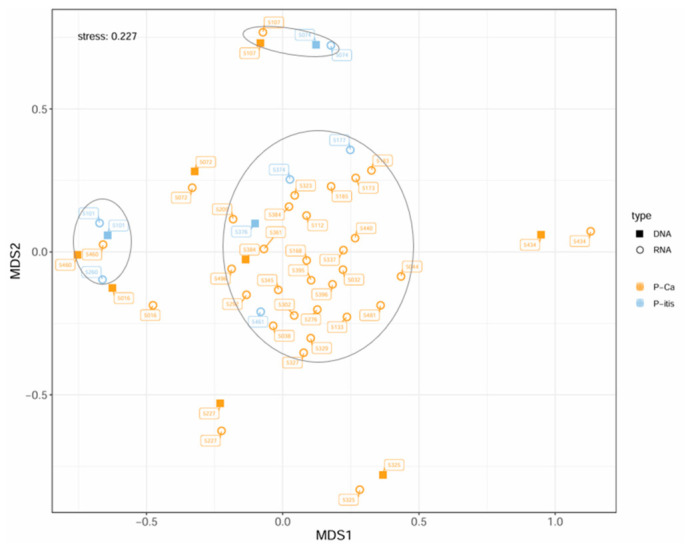

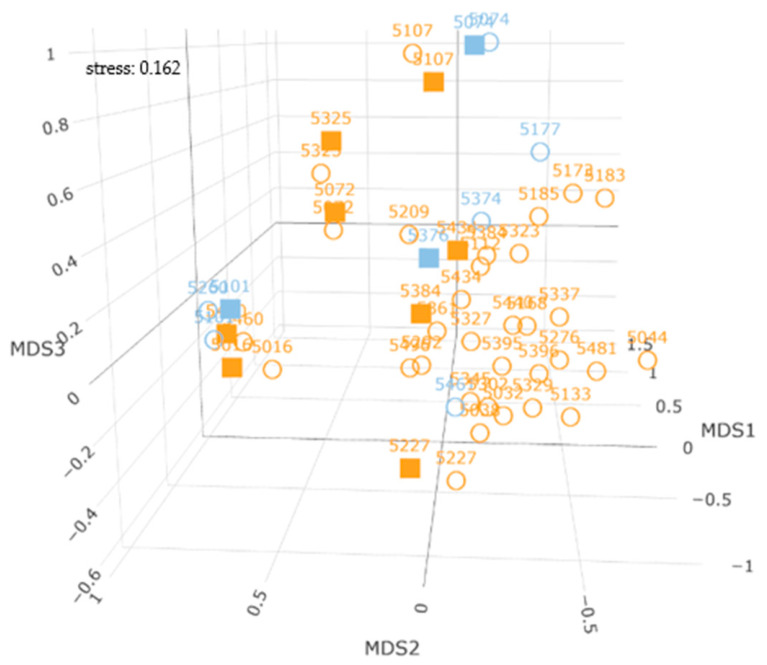
2D and 3D nMDS representation of the microbial composition of patients with pancreatic cancer and pancreatitis based on Bray–Curtis distances using only the associated pancreatic secretion samples. P-Ca= patients with pancreatic cancer; P-itis = patients with chronic pancreatitis. The labeling of the points refers to the associated patient numbers (patient ID). An existing sample clustering was outlined in grey.

Figure 6 compares the beta diversity between patients with pancreatic cancer and those with chronic pancreatitis based on pancreatic fluid samples. A total of 40 samples from 32 pancreatic cancer patients and 9 samples from 7 pancreatitis patients were analyzed.

No clear separation between the diagnostic groups was observed in the 2D nMDS plot (stress = 0.227), which improved slightly in the 3D representation (stress = 0.162). While most samples had the tendency to group centrally irrespective of diagnosis, smaller subgroups were detected. DNA- and RNA-based samples from the same patient consistently localized closely, indicating high similarity between the active and total microbial communities.

Overall, the results suggest a substantial overlap in the microbial composition of pancreatic fluid between pancreatic cancer and chronic pancreatitis patients, particularly at the RNA level, with patient-specific differences contributing to observed variability.

PERMANOVA revealed no significant differences in microbial composition between pancreatic cancer and chronic pancreatitis patients (*p* = 0.330; F = 1.087; R^2^ = 0.022). Separate analyses of DNA-based and RNA-based datasets confirmed the absence of significant differences (DNA: *p* = 0.978; R^2^ = 0.039; RNA: *p* = 0.425; R^2^ = 0.027). Including data type and its interaction with group assignment slightly increased explained variance (R^2^ = 0.064) but remained non-significant (*p* = 0.375).

While data type showed a borderline trend (*p* = 0.044; R^2^ = 0.033), the group × data type interaction was not significant (*p* = 0.999). In contrast, incorporating patient ID explained the vast majority of variance (R^2^ = 0.977; *p* < 0.001), indicating that interindividual differences dominate the variability of pancreatic secretion microbiomes rather than disease status. A detailed summary of PERMANOVA results is provided in the Supplemental Materials.

**Figure 7:**
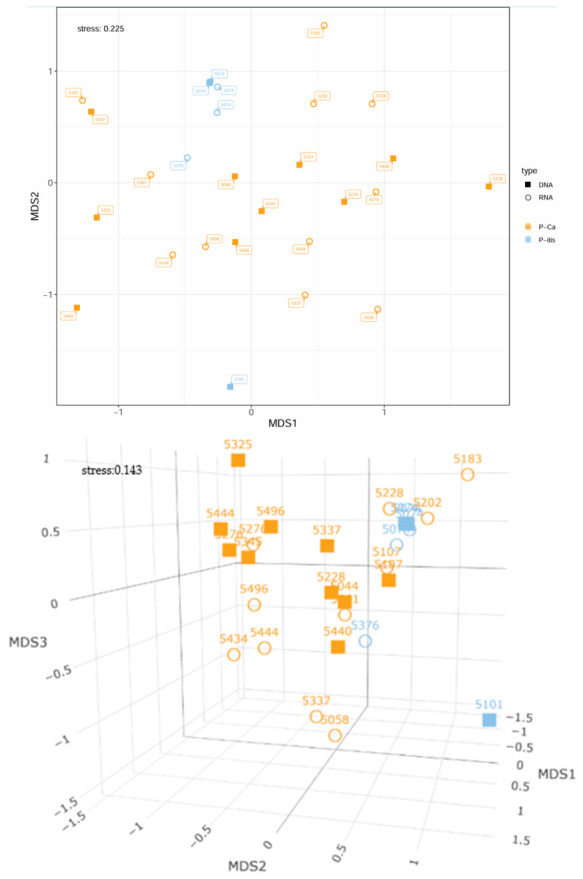
2D and 3D nMDS representation of the microbial composition of patients with pancreatic cancer and pancreatitis based on Bray–Curtis distances using only the associated tissue samples. P-Ca = patients with pancreatic cancer (C25); P-itis = patients with pancreatitis (K86.0, K86.1). The labeling of the dots refers to the associated patient numbers (patient ID). No differentiation of tissue types was made in the pancreatitis group. For this reason, patient 5074 had two samples of the same type of analysis (received as macroscopically unchanged, macroscopically altered).

If the beta diversity is compared on the basis of the tissue samples, no clear demarcation of the two diagnostic groups can be seen at first glance in Figure 7 in the 2D nMDS representation. 21 carcinoma samples from 15 patients were used. In contrast, only three pancreatitis patients with six tissue samples could be included in the graph. No differentiation of tissue types was made in the pancreatitis group. The samples from pancreatic cancer patients are distributed over the entire diagram without clustering. Even in the pancreatitis group, no clear spatial grouping can be observed. The stress value of 0.225 could be reduced to 0.143 by a 3D representation and allows a more differentiated view. There is a slight partial separation of both diagnostic groups based on the MDS2 axis. Visually, a certain similarity between the two tissue groups can be assumed, which tends to take place more at the RNA level (i.e., the active bacterial level), whereas at the DNA level the differences appear to be somewhat more pronounced.

PERMANOVA analysis revealed a significant difference between the pancreatitis group and pancreatic cancer group (*p* = 0.033; F = 1.945; R^2^ = 0.072). The low R^2^-value allows the conclusion that, despite statistically significant differences, only a small proportion of the total variation is explained by the different diagnoses. However, separate analyses of DNA-based and RNA-based datasets remained non-significant (DNA: *p* = 0.109; F = 1.454; R^2^ = 0.117; RNA: *p* = 0.535; F = 0.961; R^2^ = 0.074). If a multifactorial PERMANOVA analysis is carried out, no significant result is obtained overall (*p* = 0.213; F = 1.163; R^2^ = 0.131), but the assessment of the separate partial effects also reveals a significant difference between the two groups (*p* = 0.021; F = 1.974; R^2^ = 0.072). The data type does not seem to have a significant influence (*p* = 0.305; F = 1.123; R^2^ = 0.041) and there is also no indication of a possible interaction (*p* = 0.968; F = 0.479; R^2^ = 0.018). Overall, there seems to be a significant difference between pancreatitis and pancreatic cancer patients within the tissue samples. However, most of the variance was explained when interindividual differences were taken into account (*p* = 0.001; F = 3.466; R^2^ = 0.886). A detailed summary of PERMANOVA results is presented in the Supplemental Materials.

### 3.9. Binary Logistic Regression

Binary logistic regression was performed to evaluate associations between selected bacterial species (≥20% prevalence, ≥1% mean relative abundance) and the risk of general and infectious postoperative complications after pancreatic surgery. Models were adjusted for potential confounders (sex, age, BMI, diabetes, smoking status) and included 50 patients, as some patient information was partially missing in the retrospective data collection. Postoperative complications were classified according to the Clavien–Dindo system and categorized as severe (Clavien–Dindo ≥ III) or non-severe. Infectious complications were analyzed collectively (yes/no), with no distinction between local and systemic infections. Table 1 summarizes the infectious complications observed in this cohort, classified according to CDC criteria, whereas Table 2 shows the distribution of the Clavien–Dindo classifications. Only the period up to hospital discharge was considered. Given the exploratory nature of the analysis, no formal correction for multiple testing was applied.

Among 15 analyzed species, *Enterococcus faecalis* was the only bacterium significantly associated with a reduced risk of serious postoperative complications (Clavien-Dindo ≥ III). A one-unit increase in its relative abundance corresponded to an 11% risk reduction (OR = 0.89, 95% CI: 0.799–0.992, *p* = 0.035).

In contrast, no significant associations were found for *Escherichia*/*Shigella*, *Enterococcus faecium*, *Escherichia*/*Enterobacter*, or any other species, although some trends were observed. For infectious complications, none of the 15 species, including *Enterococcus faecalis*, showed a statistically significant effect (all *p* > 0.05).

These findings suggest a potential protective role of *Enterococcus faecalis* regarding severe postoperative morbidity, whereas the presence or abundance of other bacterial species did not significantly influence postoperative outcomes. However, a genuine effect may have been obscured by the use of relative abundances, implying that these results should be interpreted with caution and that a significant effect could indeed be present.

Further results and detailed analyses are presented in the Results section of the Supplementary Materials.

## 4. Discussion

In this study, we comprehensively characterized the microbiome of patients undergoing pancreatic surgery for malignant and chronic inflammatory diseases. Using 16S rRNA sequencing, microbial profiles were generated from 224 samples obtained from bile, pancreatic secretions, duodenum, tumor tissue, and adjacent healthy tissue across 58 patients. Our findings demonstrate that the biliary and pancreatic environment harbors a diverse and metabolically active microbiome, challenging the historic assumption of sterility in these regions. Distinct microbial communities were identified across anatomical sites, with Firmicutes, Proteobacteria, Actinobacteria, and Bacteroidetes dominating the overall composition. Importantly, differences in alpha- and beta-diversity were observed between duodenum, bile, and pancreatic secretions, while tumor and adjacent healthy tissue exhibited broadly similar microbial profiles. Additionally, we found significant associations between preoperative biliary stenting and microbial community shifts, and we identified *Enterococcus faecalis* as a species potentially associated with a lower risk of severe postoperative complications, although this result requires cautious interpretation due to limited statistical power.

The taxonomic composition observed in our cohort aligns with prior studies investigating the pancreatic and biliary microbiome in patients with PDAC and other pancreaticobiliary diseases [18,19,20,21]. In agreement with Langheinrich et al., we observed Firmicutes and Proteobacteria as the dominant phyla, while Actinobacteria and Bacteroidetes were variably represented depending on the anatomical site [18]. However, our study identified lower relative abundances of Fusobacteria (<1%) compared to previous reports that found this phylum to be more prevalent [19,20].

We also observed substantial interindividual variability in microbial profiles, consistent with findings by Del Castillo et al., who attributed the majority of variance in pancreatic and biliary microbiomes to patient-specific factors rather than disease status [19]. Interestingly, in contrast to prior studies reporting significant compositional differences between tumor and adjacent non-tumor tissues [21,22], our data revealed similar alpha- and beta-diversity patterns between these compartments.

Regarding biliary stenting, our results corroborate previous evidence suggesting that preoperative biliary drainage alters the microbial environment. Shrader et al. reported an increased bacterial load and compositional changes following stent placement, potentially influencing local inflammation and tumor progression [23]. Consistent with Blanco-Míguez et al., we found an enrichment of *Streptococcus anginosus*, *E. faecalis*, and *Klebsiella* in stented patients, indicating a predisposition toward biofilm-forming and antibiotic-resistant species [12].

Importantly, our study provides novel insights into functional differences between the total and metabolically active microbiome. RNA-based analyses revealed that in pancreatic secretions, despite low overall genetic diversity, metabolically active bacterial communities were disproportionately abundant. Such findings highlight the potential biological significance of specific active taxa, supporting emerging data suggesting that active microbial communities may modulate local immune responses and influence postoperative outcomes [24,25].

Our findings suggest that the biliary and pancreatic microbiome is highly diverse and anatomically specific, with duodenal samples demonstrating the highest alpha-diversity, while bile exhibited the lowest. Notably, RNA-based sequencing identified distinct metabolically active bacterial subsets, particularly within pancreatic secretions, underscoring the importance of distinguishing between total microbial presence and functional activity. These differences may be relevant to host–microbe interactions, local immune modulation, and susceptibility to postoperative complications.

Preoperative biliary stenting was associated with marked alterations in microbial composition, characterized by enrichment of *Streptococcus*, *Enterococcus*, and *Klebsiella*—species known to promote biofilm formation and harbor antibiotic resistance genes [12,26,27]. These findings support prior studies linking stent-related dysbiosis to an increased risk of infectious complications and poor postoperative outcomes [23,28]. In particular, patients undergoing preoperative ERCP with concomitant presence of *E. faecalis* in bile aspirates showed the highest mortality rates, indicating a possible interaction [29]. Clinically, this underscores the need for stricter indications for preoperative biliary drainage and for considering microbiome-informed perioperative antibiotic strategies.

Although diversity analyses revealed statistically significant differences, their biological impact appears limited due to the small effect sizes. In contrast, interindividual variability emerged as the predominant factor shaping microbial composition.

Regarding postoperative complications, we explored the impact of dominant taxa on severe and infectious outcomes. While no bacterial species significantly increased risk, *E. faecalis* showed a nominal association with reduced odds of severe postoperative complications (OR 0.89 per 1 percentage point increase, 95% CI 0.80–0.99, *p* = 0.035). This finding contrasts with prior literature identifying *E. faecalis* as a key contributor to surgical site infections, pancreatic fistula formation, and poor healing [29,30,31,32]. However, most observed relative abundances were far below 1%, meaning that a 1%-point increase represents an unrealistically large change compared with typical patient differences. Realistic changes correspond to minimal effects on risk. Rescaling the predictor indicates that effects corresponding to typical within-sample variation are very small (OR ≈ 0.998 per 0.01% increase), supporting a negligible practical impact. Therefore, this finding should be interpreted cautiously. Considering the relatively small cohort, the evaluation of multiple taxa without correction for multiple testing, and the use of relative abundance data, the possibility of a type I error cannot be excluded. Therefore, this finding should be regarded as exploratory and hypothesis-generating, requiring validation in larger cohorts. Nonetheless, the observation raises the intriguing possibility that some taxa may exert protective roles under certain conditions, warranting further investigation into microbiome–host interactions. However, the regression analyses considered only relative abundances, which may have obscured significant associations if changes in absolute abundances were not reflected in the relative proportions.

This study offers several methodological strengths. First, we combined DNA- and RNA-based 16S rRNA sequencing, enabling differentiation between total and metabolically active microbial communities, a rarely applied approach in pancreatic surgery. Second, we analyzed a broad range of sample types, including bile, pancreatic secretions, duodenum, tumor, and healthy tissue, providing a comprehensive view of the local microbiome. Third, standardized intraoperative sample collection minimized contamination risk, and sequencing protocols were uniformly applied across all samples, enhancing reproducibility and comparability.

However, several limitations must be acknowledged. The single-center design and modest sample size (n = 58) limit generalizability and reduce statistical power, particularly in subgroup analyses. High interindividual variability further complicated comparisons between disease states and anatomical regions. The retrospective collection of clinical data introduces potential biases related to incomplete documentation, particularly concerning preoperative stenting and postoperative complications. Additionally, the lack of a healthy control group precludes direct evaluation of disease-associated dysbiosis, and absence of longitudinal sampling limits assessment of microbiome dynamics over time. To increase statistical power for multivariable regression analyses, patients with different disease entities were pooled. This approach introduces biological heterogeneity and may confound associations between diagnosis and microbiome composition, which should be considered when interpreting group-level differences. Owing to the relatively small cohort size, adjustment in the multivariable models had to be restricted to a limited number of general clinical risk factors, which may have limited the ability to fully account for procedure-specific perioperative confounders. In addition, preoperative biliary stenting was analyzed as a binary variable, although its microbiological consequences may vary depending on stent-specific characteristics and procedural circumstances. Finally, while 16S rRNA sequencing offers robust taxonomic resolution, it cannot fully elucidate functional implications, underscoring the need for future metagenomic and metabolomic studies.

Our findings have several important clinical and research implications. First, given the observed impact of biliary stenting on microbial composition, future studies should evaluate tailored antibiotic regimens targeting dominant taxa in stented patients to reduce postoperative infections. Second, the significant differences between total and active microbial communities suggest that RNA-based profiling may better capture biologically relevant taxa, particularly when investigating host–microbe interactions and their role in complications or therapy response.

Given the single-center design and the limited cohort size, the present analysis should be considered exploratory and hypothesis-generating, warranting validation in larger, prospectively collected multicenter cohorts to overcome the limitations of small sample size and heterogeneity. Integrating shotgun metagenomic sequencing and metabolomic profiling will allow functional characterization of taxa, enabling exploration of potential microbial biomarkers for early diagnosis, prediction of complications, and personalized therapeutic strategies. Furthermore, interventional studies investigating microbiome-modifying approaches, such as targeted probiotics or selective decontamination, hold promise for reducing postoperative morbidity.

Finally, our results emphasize that pancreatic and biliary cancers, which remain associated with poor prognosis and limited treatment options, may be influenced by local microbial communities. Improved understanding of the microbiome’s role in disease progression and treatment response could provide new avenues for precision medicine in pancreatic surgery.

## 5. Conclusions

This study provides a comprehensive characterization of the pancreatic, biliary, and duodenal microbiome in patients undergoing pancreatic surgery, revealing distinct microbial compositions across anatomical sites and pancreatic diseases, with preoperative biliary stent placement significantly influencing beta-diversity. By integrating DNA- and RNA-based analyses, we demonstrate the functional relevance of metabolically active microbial communities and the added value of separate assessments for total and active microbiota. While *Enterococcus faecalis* showed a potential association with a reduced risk of severe postoperative complications, no significant impact was observed for other common taxa. Larger prospective studies are needed to validate these findings, identify clinically relevant microbial targets, and explore the microbiome’s role in pancreatic disease pathogenesis, perioperative risk stratification, and the development of future diagnostic and therapeutic strategies.

## Figures and Tables

**Table 1:** Overview of postoperative infectious complications in this study population.

	Infectious Complications	n (%)
Local	Postoperative surgical site infection (SSI) • Superficial • Deep • Organ/Space Phlegmon Urinary tract infection Nosocomial pneumonia Clostridium infection	17 (43.6%) 3 4 10 1 (2.6%) 3 (7.8%) 6 (15.4%) 2 (5.1%)
Systemic	Symptomatic COVID-19-infection Bacteremia Bloodstream infection Sepsis • Septic shock Increase of inflammatory markers with antibiotics	2 (5.1%) 1 (2.6%) 2 (5.1%) 3 (7.8%) 3 2 (5.1%)
Total		39 (100%)

**Table 2:** Distribution of Clavien–Dindo classification grades within the study population.

	0	I	II	IIIa	IIIb	IVa	IVb	V
n (%)	9 (15.5%)	16 (27.6%)	8 (13.8%)	12 (20.7%)	3 (5.2%)	6 (10.3%)	1 (1.7%)	3 (5.2%)

## Data Availability

The data presented in this study are available on request from the corresponding author due to ethical and data protection restrictions related to patient information.

## Supplementary Materials

The following supporting information can be downloaded at: https://www.mdpi.com/article/10.3390/microorganisms14050951/s1, Figure S1: 2D and 3D nMDS representation of the microbial composition of patients with pancreatic cancer and pancreatitis based on Bray-Curtis distances using only the associated tissue samples; Figure S2: 2D nMDS representation of the microbial composition of patients with pancreatic carcinoma and bile duct carcinoma based on Bray-Curtis distances using only the associated tumorous tissue samples; Figure S3: 2D and 3D nMDS representation of the microbial composition of macroscopically healthy and tumorous tissue samples in pancreatic cancer patients based on Bray-Curtis distances; Table S1: Summary of results from permutational multivariate analyses of variance (PERMANOVA) assessing microbial variance according to sample origin (duodenum vs. bile vs. pancreatic fluid), considering additional influencing factors; Table S2: Summary of permutational multivariate analyses of variance (PERMANOVA) assessing microbial variance in relation to stent status and additional potential influencing factors; Table S3: Summary of the results of permutation-based multivariate analysis of variance (PERMANOVA) for the analysis of microbial variance in secretion samples depending on the diagnosis group (pancreatic carcinoma or pancreatitis) taking into account other influencing factors; Table S4: Summary of the results of permutation-based multivariate variance analyses (PERMANOVA) for the analysis of microbial variance in tissue samples as a function of the diagnosis group (pancreatic carcinoma or pancreatitis) taking into account other influencing factors; Table S5: Summary of the results of permutation-based multivariate variance analyses (PERMANOVA) for the analysis of microbial variance in tissue samples depending on the diagnostic group (pancreatic carcinoma or bile duct carcinoma) taking into account other influencing factors; Table S6: Summary of the results of permutation-based multivariate variance analyses (PERMANOVA) for the analysis of microbial variance in pancreatic cancer patients depending on tissue status (healthy or tumorous) taking into account other influencing factors; Table S7: Summary of the non-significant results of the binary logistic regressions for bacterial species >20% and >1% excl. Escherichia/Shigella, *E. faecium*, *E. faecalis* and Escherichia/Enterobacter.

## Abbreviations

16S rRNA	16S ribosomal ribonucleic acid
BMI	Body mass index
CCA	Cholangiocarcinoma
DNA	Deoxyribonucleic acid
ERCP	Endoscopic retrograde cholangiopancreatography
FMT	Fecal microbiota transplantation
HBV	Hepatitis B virus
HCV	Hepatitis C virus
HPV	Human papillomavirus
LPS	Lipopolysaccharides
MDSCs	Myeloid-derived suppressor cells
nMDS	Non-metric multidimensional scaling
PBD	Preoperative biliary drainage
PCR	Polymerase chain reaction
PDAC	Pancreatic ductal adenocarcinoma
PERMANOVA	Permutational multivariate analysis of variance
RNA	Ribonucleic acid
TLRs	Toll-like receptors
