# Supplementary material for: Microbiome Diversity in Pancreatic Surgery: Associations with Preoperative Stenting and Postoperative Outcomes
Source: Microorganisms. 2026 Apr 23;14(5):951. doi: 10.3390/microorganisms14050951 (PMC13209569; doi:10.3390/microorganisms14050951)
Supplement: Supplementary file 1 [file microorganisms-14-00951-s001.zip › microorganisms-4217679-supplementary.pdf]

Supplementary Materials for

Microbiome Diversity in Pancreatic Surgery: Associations with Preoperative Stenting and Postoperative Outcomes

Laura Oelschlägel[1], Jörg Kleeff[1], Johannes Klose [1,] Bogusz Trojanowicz [1], Artur Rebelo[1]

[1] Department of Visceral, Vascular and Endocrine Surgery, University Hospital Halle (Saale), Martin-Luther-University Halle-Wittenberg

1. Methods

Study Material

<u>Selection of the Patient Cohort</u>

The collection and examination of patient material for research purposes were approved by the Ethics Committee of Martin Luther University Halle-Wittenberg under approval number 2019-037. Between October 2021 and April 2023, tissue and fluid samples were collected from a total of 72 patients. All patients who underwent pancreatic surgery at University Hospital Halle (Saale) during this period were included. Patients with pancreatic neoplasms, biliary tract neoplasms and chronic pancreatitis *(K86.0, K86.1)* were enrolled in the study.

<u>Sample Collection</u>

During surgery, bile fluid and pancreatic secretions were collected intraoperatively and immediately transferred into 4 mL Eppendorf tubes in the operating room. Pancreatic secretions were pipetted into a 4 mL Eppendorf tube prefilled with 1.5 mL RNA-Later stabilization solution (Invitrogen, Carlsbad, California, USA).

Tissue samples were obtained by the Department of Pathology at University Hospital Halle (Saale) during intraoperative frozen section analysis. Whenever possible, tissue samples were collected from the duodenum, macroscopically healthy tissue, and macroscopically pathological tissue.

A total of 412 samples were collected from 70 patients within the specified study period. Complete sample sets were available for 10 of these 70 patients. After collection, all tissue and fluid samples were stored at –80 °C. The frozen specimens were subsequently shipped on dry ice to the Helmholtz Centre for Infection Research in Braunschweig for further processing as described in Chapter 3.2.

Microbiome Analysis via 16S rRNA Sequencing

RNA and DNA Isolation

RNA and DNA isolation was performed using the AllPrep DNA/RNA Mini Kit (Qiagen, Hilden, North Rhine-Westphalia, Germany). First, the samples were transferred into 2 mL Lysing Matrix E tubes (MP Biomedicals, Irvine, California, USA) and lysed using 700 µL RLTplus buffer combined with 7 µL β-mercaptoethanol in a FastPrep-24™ Classic Instrument (MP Biomedicals, Irvine, California, USA) at 2 × 6.0 m/s for 45 seconds each. The samples were then centrifuged at 14,000 rpm for 10 minutes.

Subsequent steps were performed according to the manufacturer's protocol. RNA was eluted with 30–50 µL RNase-free water and DNA with 50 µL EB buffer. Complementary DNA (cDNA) synthesis was performed using the SuperScript™ IV First-Strand Synthesis System (Invitrogen, Carlsbad, California, USA) and random primers, following the manufacturer's instructions.

Amplification of the 16S rRNA Gene

To amplify the V1-V2 variable regions of the 16S rRNA gene, a three-step PCR protocol was used. Initially, the primer 27Bif (3'-AGRGTTHGATYMTG-GCTCAG-5'), optimized for amplifying Bifidobacteriaceae, and the primer 338R (5'-TGCTGCCTCCCGTAGGAGT-3') were used in the first PCR, conducted for 20 cycles to enrich the target sequences.

The second PCR used the same primers for 15 cycles, incorporating an artificial overhang that was subsequently utilized in the third PCR (10 cycles) to add both the indices and Illumina adapters to the amplicons. The amplification products were purified, normalized, and pooled using a SequalPrep™ Normalization Plate, followed by sequencing on an Illumina MiSeq™ platform (Illumina, San Diego, California, USA) with a 2 × 300 bp paired-end protocol.

From a total of 412 samples, 142 samples failed to yield usable PCR products.

Data Processing and Quality Control

After amplification, the FASTQ files were analyzed using the dada2 package (version 1.12.1) in R, generating amplicon sequence variants (ASVs). Quality trimming and filtering were performed using the "FilterAndTrim" function, removing 20 bases from the 5' end of forward reads and 19 bases from reverse reads. Reads were trimmed to a length of 240 bases, allowing a maximum of two expected errors per read.

Chimeras were removed after denoising and merging of paired-end reads. Before taxonomic classification, sample quality was assessed to ensure inclusion of only high-quality sequences. Several PCR products contained a high proportion of human sequences, and in some cases, Escherichia coli contamination from the reverse transcriptase (RT) was detected.

The number of reads affected by RT-associated E. coli contamination was quantified, and samples were evaluated accordingly. Samples with fewer than 5,000 high-quality reads were excluded. If >50% of reads were identified as E. coli, but more than 5,000 high-quality reads remained after exclusion, the sample was retained.

After quality control, 224 samples from 58 patients were included in the downstream analyses.

Amplicon Sequence Variant (ASV) Analysis and Taxonomic Classification

Amplicon sequence variants (ASVs) were assigned using a naive Bayes classifier with a pseudo-bootstrap threshold of 80% based on the Ribosomal Database Project (RDP) dataset, version 18 (RDP-Set18). Non-bacterial sequences, including eukaryotes, mitochondria, and chloroplasts, were manually removed.

Further analysis of ASVs was performed manually using the RDP database with the Seqmatch function to evaluate the reliability of each sequence type. Assignment of an ASV to a specific bacterial species was only accepted if the 16S rRNA gene fragment matched previously described isolate reference sequences with a maximum of two mismatches.

In total, 8,851 ASVs were identified and assigned to 1,624 taxa across different taxonomic levels. For certain bacterial genera, species-level classification was not possible due to low taxonomic resolution of the V1-V2 region of the 16S rRNA gene. This applied particularly to the Enterobacteriaceae family; for example, *Escherichia coli* and *Shigella* cannot be differentiated based on their 16S rRNA sequence and are thus referred to as "Escherichia/Shigella" in this study. Similar cases are consistently denoted using a slash notation.

Subsequently, for each sample, the relative abundance of detected bacterial species was calculated and used for further analyses.

Description of the Patient Cohort

Retrospective Collection of Clinical Parameters

To provide a demographic description of the study cohort, evaluate the clinical history, and assess postoperative outcomes, a set of clinical and demographic parameters was collected retrospectively using the hospital's ORBIS KIS software (Dedalus Global).

Data sources included surgical reports, pathology findings, discharge summaries, admission letters, anesthesia records, intensive care transfer notes, follow-up visits, and nursing documentation. The following information was retrieved:

- Demographics: age, sex, body mass index (BMI), ASA classification, and comorbidities
- Health-related parameters: presence of diabetes mellitus and smoking status (self-reported)
- Disease-specific data: diagnosis according to ICD-10, UICC stage, histology, tumor grading, and preoperative biliary stenting
- Surgical parameters: type and extent of resection, including extended resections such as splenectomy and/or partial hepatectomy if applicable
- Postoperative course: assessment of complications using intensive care records and discharge summaries.

Evaluation of the Microbiome Analysis

Taxonomic Classification of Bacterial Species

Based on the identified bacterial species, a complete taxonomic classification was performed using the following databases:

- Integrated Taxonomic Information System (ITIS)
- National Center for Biotechnology Information (NCBI)
- Global Biodiversity Information Facility (GBIF)

Additionally, the R package “taxize” was used to support automated annotation. For bacterial species that could not be classified automatically, a manual assignment was performed using the Human Oral Microbiome Database (HOMD) and the Metagenomic Intra-species Diversity Analysis System (MIDAS).
The taxonomic nomenclature in this study follows previously published literature to maintain consistency, even where it may deviate from the most recent international guidelines.

Group-Specific Microbiome Description

Samples were categorized according to their anatomical origin and underlying diagnosis group.

- Alpha diversity was assessed using the Shannon index, and differences between groups were tested for significance using the Kruskal-Wallis test.
- For each identified bacterial species, both the mean relative abundance and the absolute frequency were calculated.
- Absolute frequency was defined as the number of samples in which a bacterial species was detected, irrespective of its relative abundance.
- Species with a mean relative abundance ≥1% were classified taxonomically at all available levels.
- For each taxonomic level and diagnosis group, mean values were normalized to 100%.
- Based on absolute counts and relative abundances, the five most dominant bacterial species were determined for each anatomical site and diagnosis group.

Comparison of Beta Diversity Across Sample Groups

The aim of this analysis was to compare the microbial composition between different sample groups, both visually and statistically.

- Non-metric multidimensional scaling (nMDS) was used for visual representation of dissimilarities.

- Permutational multivariate analysis of variance (PERMANOVA) was applied to test for statistical significance of group differences.

Definition of Sample Groups

The grouping of samples was based on the research questions defined in **Chapter 2**. Six pairwise comparisons were defined:

- Comparison 1:
  Duodenal, bile, and pancreatic fluid samples were compared for similarity. Only patients without stents in the respective anatomical region were included to avoid bias from foreign material. Thus, bile samples from patients with biliary stents and pancreatic fluid from patients with pancreatic stents were excluded.

- Comparison 2:
  Patients with documented biliary stents were compared with patients without stents. Only bile samples were analyzed. For this comparison, patients with previous pancreatic stenting were considered "unstented," as only the biliary system was assessed.

- Comparison 3:
  To compare malignant versus inflammatory processes within the pancreas, samples from patients with pancreatic carcinoma and those with chronic pancreatitis were analyzed. Comparisons were conducted separately for pancreatic fluid and tissue samples. For pancreatic carcinoma patients, only tumor tissue samples were included.

- Comparison 4:
  To compare two malignancies, tumor tissue samples from patients with pancreatic carcinoma and cholangiocarcinoma were analyzed.

- Comparison 5:
  To compare healthy tissue with tumor tissue from the same organ system, paired macroscopically healthy and tumor tissue samples from pancreatic carcinoma patients were analyzed.

Visual Representation Using Non-Metric Multidimensional Scaling (nMDS)

The relative abundances of bacterial species within each sample were used to create a dissimilarity matrix based on Bray-Curtis distances. Using this matrix, microbial differences between samples were visualized via non-metric multidimensional scaling (nMDS) in a two-dimensional space.

Analyses were performed using the "vegan" R package (version 2024.12.1+536). Both RNA- and DNA-based datasets were included in the analyses when available for the same sample. To reduce the stress levels of the visualizations and improve interpretability, all comparisons were also visualized in a three-dimensional space.

Permutational Multivariate Analysis of Variance (PERMANOVA)

PERMANOVA was applied to assess whether, and to what extent, the microbial composition differed significantly between the predefined groups described in Chapter 3.5.1. Analyses were based on Bray-Curtis dissimilarity and performed using the "adonis2" function of the "vegan" R package (version 2024.12.1+563) with 999 permutations.

Initially, a simple model was applied to evaluate the overall effect of group membership on microbial composition across all samples. Subsequently, DNA-based and RNA-based datasets were analyzed separately to identify potential effects within each data type, using filtered datasets and separate distance matrices.

A multifactorial PERMANOVA model was additionally conducted, which included:

- The effect of group membership
- The effect of data type (DNA vs. RNA)
- The interaction effect between these two factors

To independently evaluate the contribution of each factor, analyses were complemented using the "by = margin" argument.

Because samples originated from different patients, an extended PERMANOVA model was applied that additionally incorporated patient ID as a random effect. This allowed us to assess the proportion of microbial variance attributable to interindividual differences versus biological factors.

For Comparison 1 and Comparison 5 (within-subject analyses), data were stratified by patient ID to examine intraindividual group differences. Due to insufficient data, stratification was not possible for Comparison 5 between healthy and tumorous pancreatic tissues.

A p-value ≤ 0.05 was considered statistically significant for all models. The R^2 value represents the proportion of overall microbial variance explained by the factors in the model; for example, $R^2 = 0.5$ indicates that 50% of the variance is explained.

Binary Logistic Regression

Binary logistic regression analyses were conducted using IBM SPSS Statistics (version 30, Windows). To investigate potential associations between specific bacterial species and postoperative outcomes, logistic regression models were fitted and adjusted for predefined confounders.

Due to the limited sample size, no statistical confounder selection tests were performed. Instead, confounders were chosen a priori based on clinical relevance:

- Sex
- Age
- Body mass index (BMI)

- Diabetes mellitus
- Smoking status

Two postoperative outcomes were analyzed:

1. Infectious complications
2. Severe complications (defined as Clavien-Dindo grade ≥ III) (84)

A significance threshold of $p < 0.05$ was applied. Results are reported as odds ratios (OR) with 95% confidence intervals (95% CI) and corresponding p-values.

<u>Operationalization of the Microbiome</u>

To evaluate whether the presence of specific bacterial species influenced postoperative outcomes, a subset of species was selected from the 1,624 identified taxa to be included as independent variables in statistical analyses.

The selection criteria were:

- Present in ≥20% of all samples
- Average relative abundance ≥1%

For each patient, the mean relative abundance across all available sample types was calculated for each selected species, resulting in a single aggregated species value per individual.

<u>Operationalization of Clinical Parameters</u>

Body Mass Index (BMI):

For demographic characterization, each patient was categorized into weight classes according to the guidelines of the German Obesity Society.
However, for logistic regression analyses, the raw BMI value was used as a continuous confounding variable.

Smoking Status:

For the smoking status of patients, only the information available at the time of surgery was considered, as the data were obtained exclusively from self-reports in anesthesia consent forms, which lacked standardized documentation.

There were no consistent records regarding the number of pack-years or the duration of smoking cessation. Patients who self-reported as former smokers were classified within the group of non-smokers; therefore, no distinction was made between former and never-smokers.

For statistical analyses, patients were categorized into two groups:

- Active smokers
- Non-smokers

Diabetes Mellitus:

Regarding diabetes status, no distinction was made between diabetes types. The presence of any diagnosis of diabetes mellitus prior to the hospital admission and surgery was considered sufficient for classification as diabetic.

Postoperative Complications:

To operationalize postoperative complications as a binary dependent variable (outcome), each patient was classified according to the Clavien-Dindo classification (see Table 3). Based on this system, patients were divided into two categories according to the severity of complications:

- Severe complications: defined as Clavien-Dindo grade ≥ III
- Non-severe complications: Clavien-Dindo grade < III

A specific focus was placed on postoperative infectious complications. No distinction was made between local and systemic infections. Consequently, patients were dichotomized according to the presence or absence of postoperative infectious complications (yes/no).

Validation of the 16S rRNA Sequencing

To assess the validity of the 16S rRNA sequencing results, the agreement between DNA-based and RNA-based sequencing outcomes and the results of intraoperatively obtained microbiological cultures was evaluated.

- For bile duct swabs, the corresponding bile fluid samples from the 16S rRNA sequencing were used for comparison.
- For pancreatic duct swabs, the corresponding pancreatic fluid samples were used.

Sensitivity was calculated at both the patient level and the species level:

- If at least one bacterial species identified in culture was not detected by sequencing, the case was classified as false negative.
- If all cultured species were detected by sequencing, the case was classified as true positive.

At the species level, each cultured bacterial isolate was compared individually:

- If a species identified by culture was also detected by sequencing, it was considered true positive.
- If the species was not detected by sequencing, it was classified as false negative.

2. Results

Characterization of the Study Cohort

Demographic Variables

Age and Sex:
A total of 58 patients were included in this study, comprising 32 males (55.2%) and 26 females (44.8%). The overall mean age was 64.1 years (SD 13.0; range 34–84). When stratified by sex, the mean age was 58.8 years for males (SD 12.6; range 34–77) and 70.7 years for females (SD 10.4; range 39–84). An age-group analysis revealed that female patients were significantly older than male patients (p=0.001, Chi-square test).

Body Mass Index (BMI):
According to the guidelines of the German Obesity Society (83), 30 patients (51.7%) had a normal BMI, while 3 patients (5.2%) were underweight. 17 patients (29.3%) were classified as obese: 13 with class I obesity (22.4%), 3 with class II (5.2%), and 1 with class III (1.7%). An additional 8 patients (13.8%) fell into the pre-obese category. Sex-stratified analysis showed that obesity was more frequent among females, whereas males predominated in the lower BMI categories. However, there was no statistically significant association between BMI category and sex (p=0.472).

Comorbidities (Diabetes Mellitus, Smoking):
At least one comorbidity was documented in 52 patients (89.7%). Diabetes mellitus was present in 15 patients (25.9%) prior to hospital admission. Only 6 patients (10.3%) had no recorded comorbidities. Smoking status was available for 50 patients: 17 patients (29.3%) were current smokers, 33 (56.9%) were non-smokers, and 5 patients (8.6%) were former smokers.

ASA Score:
Based on the American Society of Anesthesiologists (ASA) classification, 52 patients (89.7%) were classified as ASA III, while 6 patients (10.3%) were classified as ASA II. No patients were classified as ASA I, IV, or higher.

Disease-Related Variables:

Histopathological diagnoses were classified according to the ICD-10 system.

- Pancreatic malignancies were the most common, occurring in 39 patients (67.1%). Among these, 31 tumors were located in the pancreatic head (C25.0), 2 in the body (C25.1), and 6 were unspecified (C25.9).
- Biliary tract malignancies were diagnosed in 11 patients (18.9%): 6 extrahepatic bile duct carcinomas (C24.0), 4 ampullary carcinomas (C24.1), and 1 overlapping lesion (C24.8).

- Chronic pancreatitis was present in 7 patients (12%) (K86.1; K86.18), with only 1 alcohol-induced case (K86.0).
- In one patient, a suspected malignancy was not confirmed (D13.6).

Biliary Tract Cancer

UICC Stage:
Among the 11 patients with biliary tract carcinomas, no predominant UICC stage was identified; all stages except IA were represented. However, 8 of 11 patients (72.8%) presented with advanced disease (≥IIB) involving lymph node metastases.

Tumor Differentiation:
Poorly differentiated tumors (G3) predominated (n=9; 81.8%). All extrahepatic bile duct carcinomas (C24.0) were poorly differentiated, whereas ampullary carcinomas (C24.1) were either moderately or poorly differentiated.

Pancreatic Cancer

UICC Stage:
Among patients with pancreatic cancer (n=39), UICC stage III was most common (35.9%), followed by stage IIB (33.3%). Overall, 87.1% of patients presented with advanced disease (≥IIB) at the time of surgery, irrespective of tumor location.

Tumor Differentiation:
Poor differentiation was also predominant among pancreatic carcinomas: G3 tumors accounted for 61.5%, G2 for 25.6%, and G1 or low-grade dysplasia for only 2.6%.

Preoperative Stent Placement

Preoperative biliary or pancreatic stent placement was documented in 32 patients (55.2%). Of these, 28 patients (87.5%) had biliary stents, 1 patient (3.1%) had a pancreatic duct stent, and 3 patients (9.4%) had both biliary and pancreatic stents. No stent placement was performed in the remaining 26 patients (44.8%).

Surgery-Related Variables:

Type of Surgery:
Surgical procedures varied depending on tumor location and histology:

- Pylorus-preserving partial pancreatoduodenectomy (PPPD): 41 cases (70.7%)
- Partial pancreatoduodenectomy (PPD, Whipple): 6 cases (10.3%)

- Total pancreatoduodenectomy (TD): 7 cases (12%)
- Distal pancreatectomy (PL): 2 cases (3.4%)
- Duodenum-preserving pancreatic head resection (DEPKR): 2 cases (3.4%)
- Tru-cut biopsy only: 1 case (1.7%)
-

Extent of Resection:
Extended resections were necessary in 22 patients (37.9%), mainly involving liver resections (n=10) and splenectomies (n=2).

Resection Status (R Classification):
Among patients undergoing surgery for malignant disease (n=50), R0 resection was achieved in 33 patients (66.0%), while 15 patients (30.0%) underwent R1 resection. In two cases (4.0%), resection status was not documented.

Postoperative Complications

Postoperative outcomes were classified according to the Clavien-Dindo system:

- No complications: 9 patients (15.5%)
- Mild complications (≤II): 24 patients (41.4%)
- Severe complications (≥III): 25 patients (43.1%)
-

Infectious complications occurred in 24 patients (41.4%), totaling 39 postoperative infectious events. Surgical site infections (SSI) were most common (17 cases; 43.6%), classified according to CDC criteria (87,88). Nosocomial pneumonia was diagnosed in 6 patients (15.4%).

A significant association was observed between infectious complications and severe postoperative morbidity (χ^2(df=1) =21.71; $p<0.001$): patients with infections were more likely to develop severe complications (79.2%) compared to those without infections (17.6%).

Description of the Microbial Composition of the Entire Sample Set

Microbiome analysis was performed using 16S rRNA gene sequencing. Amplicon sequence variants (ASVs) were taxonomically classified using a naive Bayes classifier in combination with the RDP database (version 18). In total, 1,623 bacterial taxa across different taxonomic levels were identified in at least one of the 224 analyzed samples. Complete species-level classification was not possible for all ASVs: 828 unique species (51.0%) were assigned at species level, while 603 taxa (37.2%) could only be classified at the genus level.

Among the identified phyla, Firmicutes represented the largest proportion (27.8%), with the dominant classes being Bacilli (43.5%), Clostridia (42.8%), and Negativicutes (8.0%). The phylum

Proteobacteria was similarly prevalent (26.9%), primarily composed of the classes Gammaproteobacteria (36.2%), Alphaproteobacteria (35.6%), and Betaproteobacteria (18.6%). Actinobacteria (22.3%) and Bacteroidetes (12.6%) were also common. Within the phylum Actinobacteria, the class Actinobacteria accounted for the vast majority (93.9%). Within Bacteroidetes, the most abundant classes were Bacteroidia (57.6%), Flavobacteria (16.1%), Sphingobacteriia (9.8%), and Cytophagia (9.8%).

Abundance and Prevalence of Bacterial Species

Despite the high taxonomic diversity, only a limited number of taxa were prevalent across multiple samples. Specifically, only 53 taxa were detected in ≥20% of all. The genus Escherichia/Shigella was the most frequently identified, detected in 183 of 224 samples (81.7%), making it the most common taxon in the dataset.

However, high relative abundance within individual samples was rare. Across all samples, only 45 bacterial species reached a relative abundance ≥10% in at least one sample When averaged across the entire cohort, only 17 species achieved a mean relative abundance ≥1%. Among these, Escherichia/Shigella again ranked highest, with an average relative abundance of 11.2%.

The bacterial species detected in ≥20% of all samples and showing a mean relative abundance ≥1% formed the basis for the binary logistic regression analyses described in Section 4.5.

Taxonomic Patterns Among the 15 Key Species

Taxonomic analysis of these 15 frequently detected species revealed patterns consistent with the overall microbial composition:

- Firmicutes was the most dominant phylum, comprising 53.3% (n=8) of these species, primarily belonging to the classes Bacilli (n=7; 87.5%) and Clostridia (n=1; 12.5%).
- Proteobacteria accounted for 40% (n=6) of the species, with all members belonging to the class Gammaproteobacteria.
- The remaining 6.7% (n=1) was represented by Bifidobacterium animalis, the only species from the phylum Actinobacteria.

PERMANOVA Analysis of sample origin

Table S1 Summary of results from permutational multivariate analyses of variance (PERMANOVA) assessing microbial variance according to sample origin (duodenum vs. bile vs. pancreatic fluid), considering additional influencing factors.

**Analysis**	**p-value**	**F-value**	**R^2**
**Group effect**			
All samples *(n = 105)*	**0.006**	1.654	0.031
DNA-based samples *(n = 36)*	0.828	0.818	0.047
RNA-based samples *(n = 69)*	0.051	1.319	0.038
**Multifactorial analysis**			
Overall effect	**0.031**	1.268	0.060
Group	0.019	1.483	0.028
Data type (DNA vs. RNA)	0.021	1.704	0.016
Interaction (Group × Data type)	0.989	0.668	0.013
**Full model incl. Patient ID**			
Overall effect	**0.001**	2.799	0.751
Group	0.004	1.866	0.018
Data type	0.522	0.931	0.005
Patient ID	**0.001**	2.915	0.698
**Intraindividual group effect** *(n = 8)* — Duodenum vs. Pancreatic fluid			
Overall effect	**0.003**	1.810	0.047
Group	0.017	1.890	0.025
Data type	0.197	1.262	0.017
**Intraindividual group effect** *(n = 16)* — Bile vs. Pancreatic fluid			
Overall effect	**0.041**	1.479	0.033
Group	0.632	0.972	0.011

Analysis	p-value	F-value	R^2
Data type	0.016	1.980	0.022

PERMANOVA Analysis of Bile Samples (Stent vs. No-Stent Groups)

Table S2. Summary of permutational multivariate analyses of variance (PERMANOVA) assessing microbial variance in relation to stent status and additional potential influencing factors.

**Analysis**	**p-value**	**F-value**	R^2
**Group effect**			
All samples *(n = 88)*	**0.001**	1.456	0.041
DNA-based samples *(n = 40)*	**0.040**	1.702	0.043
RNA-based samples *(n = 48)*	**0.001**	2.637	0.054
**Multifactorial analysis**			
Overall effect	**0.001**	1.915	0.064
Group (stent vs. no stent)	**0.001**	3.561	0.039
Data type (DNA vs. RNA)	0.190	1.267	0.014
Interaction (Group × Data type)	0.726	0.795	0.008
**Full model incl. Patient ID**	**0.001**	5.649	0.889

PERMANOVA Analysis of Beta Diversity Between Patients With Pancreatic Cancer and Chronic Pancreatitis

Table S3: Summary of the results of permutation-based multivariate analysis of variance (PERMANOVA) for the analysis of microbial variance in secretion samples depending on the diagnosis group (pancreatic carcinoma or pancreatitis) taking into account other influencing factors

	p-value	*F-value*	*R2 value*
Group Effect			
all samples (n=49)	0,330	1,087	0,022
DNA samples only (n=11)	0,978	0,367	0,039
RNA samples only (n=38)	0,425	1,002	0,027
Multifaktorial Analysis			
Overall effect	0,375	1,036	0,064
Group	0,429	1,001	0,021
Data type	**0,044**	1,656	0,033
Interaction	0,999	0,388	0,008
Overall model incl. PatientID	**0,001**	10,015	0,977

<u>Tissue</u>

If the beta diversity is compared on the basis of the tissue samples, no clear demarcation of the two diagnostic groups can be seen at first glance in Figure S1 in the 2D nMDS representation. 21 carcinoma samples from 15 patients were used. In contrast, only three pancreatitis patients with six tissue samples could be included in the graph. No differentiation of tissue types was made in the pancreatitis group. For this reason, patient 5074 had two samples of the same type of analysis (received as macroscopically unchanged, macroscopically altered) that were in close proximity to each other. The samples from pancreatic cancer patients are distributed over the entire diagram without clustering. Even in the pancreatitis group, no clear spatial grouping can be observed. The stress value of 0.225 could be reduced to 0.143 by a 3D representation. The 3D-nMDS thus allows a more differentiated view. There is a slight partial separation of both diagnostic groups based on the MDS2 axis. While pancreatitis samples are exclusively in the MDS2-positive range, pancreatic cancer samples are more scattered, but predominantly in the MDS2-negative range. In addition, a closer spatial location of the RNA samples compared to the DNA samples can be guessed. Visually, a certain

similarity between the two tissue groups can be assumed, which tends to take place more at the RNA level (i.e. the active bacterial level), whereas at the DNA level the differences appear to be somewhat more pronounced.

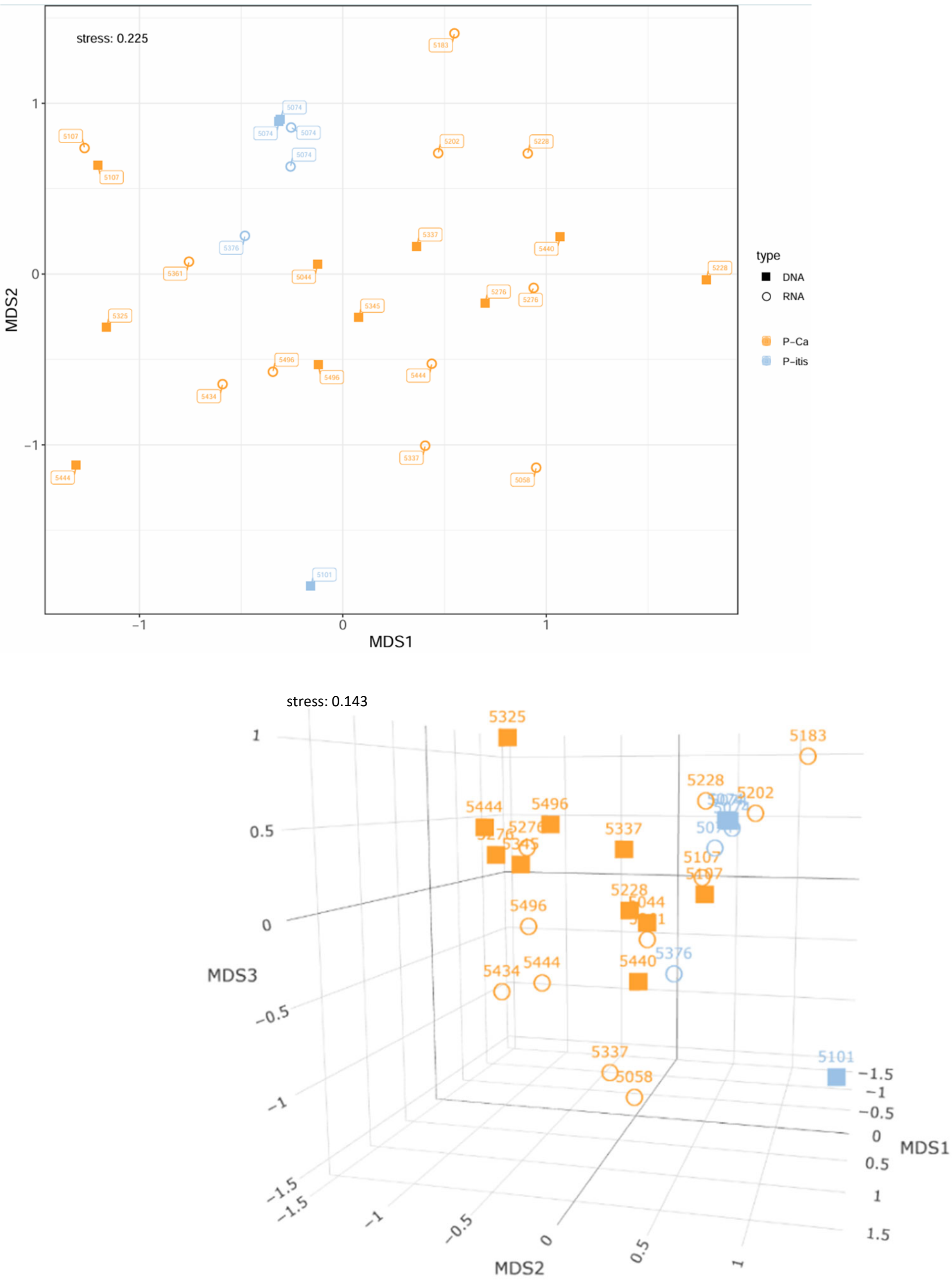

Figure S1: 2D and 3D nMDS representation of the microbial composition of patients with pancreatic cancer and pancreatitis based on Bray-Curtis distances using only the associated tissue samples

P-Ca= patients with pancreatic cancer (C25); P-itis = patients with pancreatitis (K86.0, K86.1).

The labeling of the dots refers to the associated patient numbers (patient ID)

PERMANOVA analysis revealed a significant difference between the pancreatitis group and pancreatic cancer group (p=0.033; F=1.945; R2 value=0.072). The low R2 value allows the conclusion

that, despite statistically significant differences, only a small proportion of the total variation is explained by the different diagnoses. If the DNA and RNA analyses are considered separately, both within the DNA samples (p=0.109; F=1.454; R2=0.117), as well as among the RNA samples (p=0.535; F=0.961; R2=0.074). If a PERMANOVA analysis is carried out, which takes into account not only the factor of the different diagnostic groups, but also the different data types and a possible interaction of both variables, no significant result is obtained overall (p=0.213; F=1.163; R2=0.131), but the assessment of the separate partial effects also reveals a significant difference between the two groups (p=0.021; F=1.974; R2=0.072). The data type does not seem to have a significant influence (p=0.305; F=1.123; R2=0.041) and there is also no indication of a possible interaction (p=0.968; F=0.479; R2=0.018). The largest increase in the R2 value and thus the highest proportion of variance could also be achieved here by adding the different patients (p=0.001; F=3.466; R2=0.886). Overall, there seems to be a significant difference between pancreatitis and pancreatic cancer patients within the tissue samples. However, most of the variance is also explained by the interindividual differences. A summary of the PERMANOVA results is presented in Table 4.

Table S4: Summary of the results of permutation-based multivariate variance analyses (PERMANOVA) for the analysis of microbial variance in tissue samples as a function of the diagnosis group (pancreatic carcinoma or pancreatitis) taking into account other influencing factors

	p-value	*F-value*	*R2 value*
Group Effect			
all samples (n=27)	**0,033**	1,945	0,072
DNA samples only (n=13)	0,109	1,454	0,117
RNA samples only (n=14)	0,535	0,961	0,074
Multifaktorial Analysis			
Overall effect	0,213	1,163	0,131
Group	**0,021**	1,974	0,072
Data type	0,305	1,123	0,041
Interaction	0,968	0,479	0,018
Overall model incl. PatientID	**0,001**	3,466	0,886

Comparison of beta diversity between patients with pancreatic and bile duct carcinomas

Figure S2 shows the beta diversity comparison between pancreatic cancer and bile duct cancer patients. For this diagram, only macroscopically tumorous tissue samples were used. 15 pancreatic cancer patients with 21 tissue samples and six bile duct cancer patients with ten tissue samples were included in the 2D-nMDS image. The samples of both diagnostic groups are relatively scattered across the diagram. Neither a clear separation of the two diagnoses nor a clear sample clustering can be determined. However, the majority of bile duct cancer samples are more likely to be in the MDS2 positive range, while the samples from pancreatic cancer patients are more likely to be found in the MDS2 negative range. However, this division is minimal. DNA and RNA analysis of the same sample are usually relatively close to each other. The original stress value of the 2D nMDS display could be reduced from 0.245 to 0.160 by means of a 3D display. Even when viewed in three-dimensional space, no separation of the two groups can be observed. Instead, the samples from bile duct and pancreatic cancer patients are distributed relatively diffusely in the room. An overlap of the DNA and RNA samples is also recognizable. Visually, a similarity of the microbial profiles within the tumorous tissue samples of both types of carcinoma can be assumed on this basis.

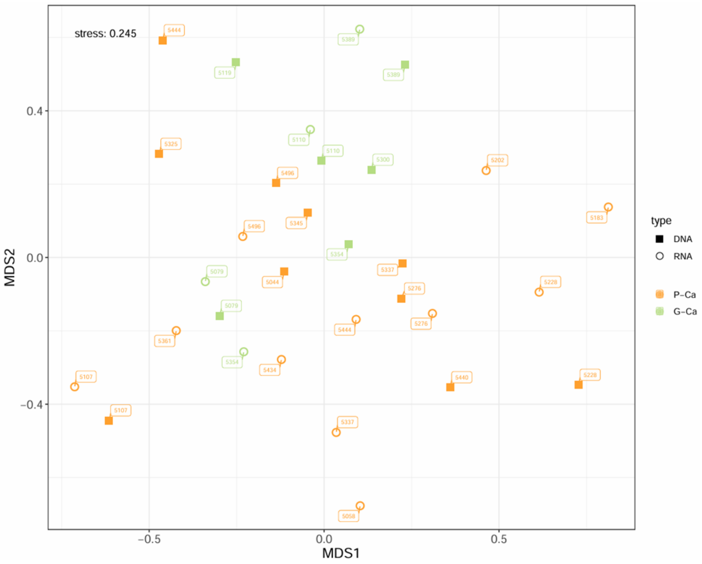

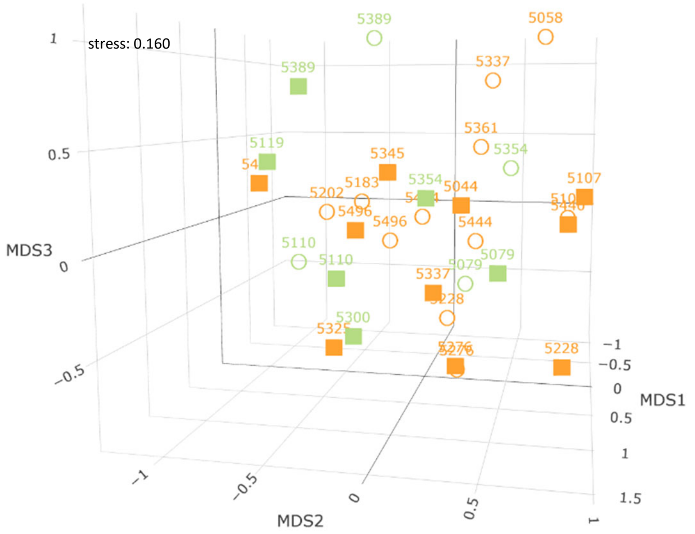

Figure S2: 2D nMDS representation of the microbial composition of patients with pancreatic carcinoma and bile duct carcinoma based on Bray-Curtis distances using only the associated tumorous tissue samples

P-Ca= patients with pancreatic cancer; G-Ca = patients with bile duct carcinoma.

The labeling of the points refers to the associated patient numbers (patient ID).

The PERMANOVA analysis also did not reveal a significant difference between the two groups (p=0.180; F=1.248; R2=0.041). Even when looking at DNA and RNA alone, no reduction in the p-value could be achieved (DNA: p=0.755; F=0.757; R2=0.051) (RNA: p=0.576; F=0.918; R2=0.066). If one takes into account the possible influences of the different data types and the possible interaction between diagnosis and data type in addition to the pure group membership, there is also no significant difference in the sample community. This results in only a slight increase in the R2 value (p=0.519; F=0.963; R2= 0.097). The use of the by=margin function to assess the individual partial effects also did not reveal any significant differences. Thus, neither the sole assignment to one of the two diagnostic groups (p=0.268; F=1.170; R2=0.038), nor does it belong to one of the two types of analysis (p=0.278; F=1.146; R2=0.038). There was also no interaction between data type and diagnosis (p=0.962; F=0.530; R2=0.018). Only the addition of the patient variable resulted in a significant difference and a significant increase in the explainable proportion of variance, which suggests a large influence of the interindividual differences (p=0.001; F=2.347; R2= 0.846). Overall, however, there is no indication that the tumorous tissue samples differ significantly in their microbial composition. Instead, other patient-specific factors seem to explain much of the microbiome differences. A summary of the PERMANOVA results is presented in Table 5.

Table S5: Summary of the results of permutation-based multivariate variance analyses (PERMANOVA) for the analysis of microbial variance in tissue samples depending on the diagnostic group (pancreatic carcinoma or bile duct carcinoma) taking into account other influencing factors

	p-value	*F-value*	*R2 value*
Group Effect			
all samples (n=31)	0,180	1,248	0,041
DNA samples only (n=16)	0,755	0,757	0,051
RNA samples only (n=15)	0,576	0,918	0,066
Multifaktorial Analysis			
Overall effect	0,519	0,963	0,097
Group	0,268	1,170	0,038
Data type	0,278	1,146	0,038
Interaction	0,962	0,530	0,018
Overall model incl. PatientID	**0,001**	2,347	0,846

Comparison of beta diversity between healthy and tumourous tissue samples in pancreatic cancer patients

In the 2D-nMDS image (Figure S3), the tumorous tissue samples are compared with the macroscopically inconspicuous tissue samples in their microbial composition. 21 macroscopically modified tissue samples and 13 healthy tissue samples from a total of 19 patients could be used. However, only five patients (5107,5183, 5361, 5440, 5444) had both types of tissue available for an internal patient comparison. Only in the samples of patient 5107 can a close spatial relationship between tumorous and healthy tissue samples be determined. In the remaining 4 patients, there are sometimes large distances between the tissue samples. In general, no clear separation of the two sample types can be determined. There is also no clear clustering. The two sample types are distributed almost evenly on the diagram. The stress value of the 2D nMDS display is 0.234, and if a three-dimensional representation is used, the stress value can be reduced to 0.162. By adding a third level, it also becomes clear that the DNA and RNA samples of the healthy tissue tend to be distributed in the MDS1-negative range. The DNA samples of the tumorous tissues, on the other hand, are preferably in the MDS2-negative range. This also applies to the RNA samples of the pathological

tissue. However, this situation is less pronounced here and there is a stronger grouping in the MDS1-positive range. A mixing of the sample types at the analysis level can be observed more strongly in the RNA samples than in the DNA samples, which suggests a stronger match of the active community (RNA). Visually, a similarity of both groups can thus be observed, especially at the RNA level. However, the distance between tumorous and healthy tissue of a patient cannot be described uniformly.

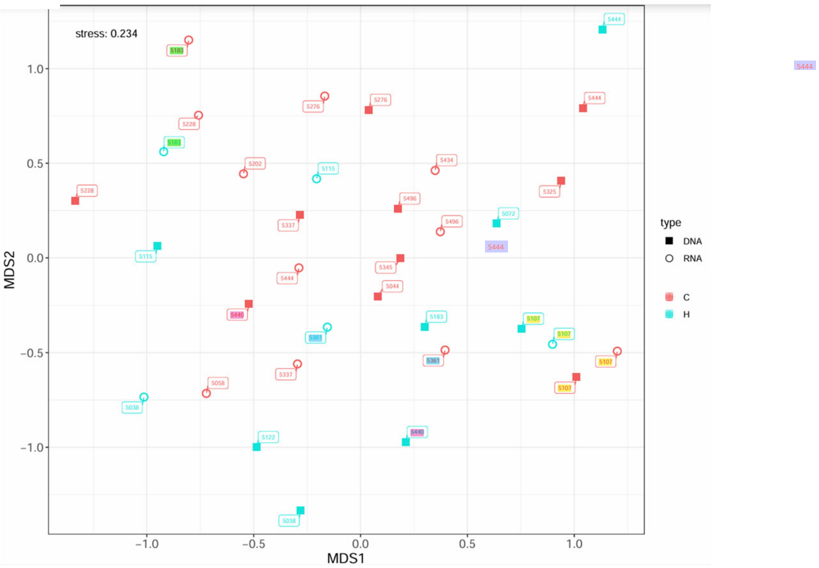

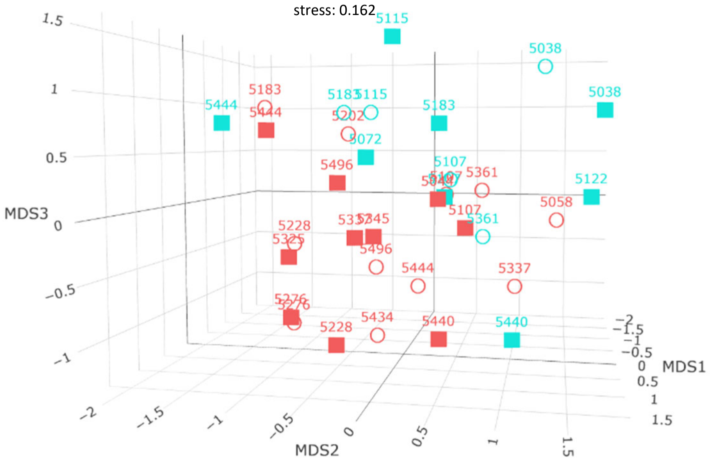

Figure S3: 2D and 3D nMDS representation of the microbial composition of macroscopically healthy and tumorous tissue samples in pancreatic cancer patients based on Bray-Curtis distances

C= cancer/tumorous tissue; H=healthy/healthy tissue.

The labeling of the points refers to the associated patient numbers (patient ID).

In the 2D representation, patients in whom both sample types (H and C) were present were marked in color.

With the help of the PERMANOVA analysis, no significant difference between the two tissue types could be observed (p=0.113; F=1.356; R2=0.041). This could also be determined when both data types were considered separately (DNA: p=0.263; F=1.183; R2=0.068) (RNA: p=0.844; F=0.668; R2= 0.046). The additional consideration of data type (RNA vs. DNA) and the possible interaction between group and data type also did not lead to an increase in significance. However, a slight increase in the R2 value from 4.1% to 9.8% was observed (p=0.284; F=1.081; R2=0.098). The individual variables also did not lead to any significant differences. Nor could an interaction be detected (p=0.987; F=0.517; R2=0.016). In another model, the influence of the different patients was included as a variable in the analysis. The addition resulted in a significant difference in the sample community (p=0.001; F=2.793; R2=0.811). If the variables are considered separately, it becomes clear that the significance and the majority of the total microbial variance seems to be explainable by the interindividual differences alone (p=0.001; F=2.79; R2=0.729). A significant difference due to the assignment to healthy and tumorous tissue cannot be detected (p=0.978; F=0.515; R2=0.007). The data type also does not significantly distinguish the samples (p=0.070; F=1.441; R2= 0.021). Overall, it can be assumed that the healthy and tumorous tissue samples do not differ significantly in their microbial composition. How both types of tissue behave within the same patient could not be investigated by stratification due to the limited data available. However, there are indications that interindividual differences in particular define the majority of the variance of this sample community. A summary of the PERMANOVA results is presented in Table 6.

Table S6: Summary of the results of permutation-based multivariate variance analyses (PERMANOVA) for the analysis of microbial variance in pancreatic cancer patients depending on tissue status (healthy or tumorous) taking into account other influencing factors

	p-value	*F-value*	*R2 value*
Group Effect			
all samples (n=34)	0,113	1,356	0,041
DNA samples only (n=18)	0,263	1,183	0,068
RNA samples only (n=16)	0,844	0,668	0,046
Multifaktorial Analysis			
Overall effect	0,284	1,081	0,098
Group	0,143	1,346	0,039

Data type	0,111	1,395	0,041
Interaction	0,987	0,517	0,016
Overall model incl. PatientID			
Overall effect	**0,001**	2,793	0,811
Group	0,978	0,515	0,007
Data type	0,070	1,441	0,021
PatientID	**0,001**	2,790	0,729

Binary logistic regression

With the help of binary logistic regression, a possible association between bacterial species and the occurrence of serious and infectious postoperative complications was investigated. For this purpose, all species that were present in ≥ 20% of all samples and at the same time achieved an average relative abundance of ≥1% were included in the statistical analysis. For each selected bacterial species, a separate binary logistic regression occurred. In addition, five confounder variables were included in each logistic regression (sex, age in years, BMI in kg/^{m2}, diabetes, smoking status). Since no smoking status could be determined for eight patients, these patients were excluded from the following analyses. For this reason, the binary logistic regressions were only performed with 50 patients. The species *Escherichia/Shigella*, *Enterococcus faecalis*, *Enterococcus faecium* and *Escherichia/Enterobacter* are among the most common pathogens of nosocomial infections. They are also considered part of the physiological intestinal flora and easily get into sterile compartments during visceral surgery. As opportunistic pathogens, they are common pathogens of postoperative infections. Their antibiotic resistance, some of which is pronounced, also makes adequate therapy difficult.

<u>Outcome defined as a serious complication according to Clavien-Dindo</u>

15 bacterial species have been identified according to the above criteria, whose influence on the occurrence of serious postoperative complications after pancreatic surgery will be investigated. Complications that fell into category III or higher according to the Clavien-Dindo classification were considered serious. The results of all logistic regressions carried out are summarized in Table 7.

Escherichia/Shigella

The investigation of an influence of the bacterial species *Escherichia/Shigella* on the development of a serious postoperative complication showed no significant effect and an odds ratio of 1.01 (95% CI: 0.979-1.035; p=0.645), which was adjusted for potential confounders.

Enterococcus faecalis

If the relationship between the occurrence of the bacterial species *Enterococcus faecalis* and the occurrence of serious postoperative complications is investigated, a significant influence can be detected with the help of binary logistic regression. If the relative abundance of the species increases by one unit, a risk reduction of 0.89 times can be observed (95% CI: 0.799-0.992; p=0.035). This effect is significant at a significance level of p=<0.05 and a confidence interval outside 1. This result is adjusted for potential confounders.

Enterococcus faecium

If binary logistic regression is used to investigate an association between Enterococcus faecalis and the occurrence of serious postoperative complications, there is no significant effect. With an odds ratio of 1.11 (95% CI: 0.937-1.314; p=0.228), only a tendency can be observed. This result indicates that the risk could increase by 1.11 times if the relative abundance of *Enterococcus faecium* increases by one unit while adjusting for potential confounders. However, this tendency is not meaningful due to a lack of significance.

Escherichia/Enterobacter

The performance of a binary logistic regression to analyze a possible association between the occurrence of the bacterial species *Escherichia/Enterobacter* and the occurrence of serious postoperative complications did not show any significant influence when adjusting for potential confounders. With an odds ratio of 1.10 (95% CI (0.889-1.265; p=0.463), a slight increase in risk of 1.10-fold can be assumed with an increase in the relative abundance of the species by one unit. However, due to the lack of significance, this result can only be judged as a tendency.

<u>Outcome defined as the occurrence of infectious complications</u>

On the basis of the 15 bacterial species identified according to the above criteria, the association between these bacterial species and the occurrence of infectious complications after pancreatic surgery was investigated.

Escherichia/Shigella

The occurrence of Escherichia/Shigella and the possible effect on the development of infectious postoperative complications was investigated using binary logistic regression. The model was likewise checked for potential confounders. The binary logistic regression resulted in an odds ratio of 0.98 (95% CI: 0.951-1.010; p=0.197). The 95% confidence interval and the p-value show no significant effect.

Enterococcus faecalis

Looking at the possible association between the occurrence of *Enterococcus faecalis* and the occurrence of postoperative infectious complications, no significant influence can be found when adjusting for potential confounders. The odds ratio in this case was 1.09 (95% CI: 0.985-1.201; p=0.098).

Enterococcus faecium

A potential association between the occurrence of *Enterococcus faecium* and the occurrence of postoperative infectious complications was investigated using binary logistic regression and the possible effect for potential confounders was adjusted. The odds ratio of 0.94 (95% CI: 0.846-1.041; p=0.232) did not result in a significant association.

Escherichia/Enterobacter

If a possible association between the occurrence of postoperative infectious complications and the occurrence of the bacterial species *Escherichia/Enterobacter* is investigated using binary logistic regression and control for possible confounders, no significant association can be found (OR=0.96 ; 95% CI:0.807-1.131; p=0.593).

Table S7: Summary of the non-significant results of the binary logistic regressions for bacterial species >20% and >1% excl. Escherichia/Shigella, E.faecium, E. faecalis and Escherichia/Enterobacter

	Serious complication (Clavien-Dindo ≥III); n=22		*Infectious complication; n=20*	
	OR (95%-KI)	*p- Wert*	*OR (95%-KI)*	*p-Wert*
Streptococcus anginosus	1,00 (0,960-1,039)	0,932	1,01 (0,971-1,053)	0,582
Klebsiella	0,97 (0,925-1,019)	0,236	1,02 (0,974-1,065)	0,416
Staphylococcus aureus	1,02 (0,988-1,059)	0,207	1,00 (0,968-1,027)	0,850

Staphylococcus epidermidis	0,76 (0,494-1,169)	0,212	0,98 (0,896-1,069)	0,631
Bifidobacterium animalis	0,68 (0,377-1,210)	0,187	1,04 (0,918-1,180)	0,533
Enterobacter/Kosakonia/ Yokanella	1,02 (0,957-1,095)	0,498	0,88 (0,741-1,064)	0,197
Streptococcus salivarius	0,86 (0,614-1,199)	0,369	1,10 (0,862-1,398)	0,447
Hafnia alvei parahalvei	0,57 (0,094-3,432)	0,539	0,78 (0,188-3,214)	0,727
Citrobacter koseri/Salmonella	1,01 (0,835-1,216)	0,936	0,97 (0,801-1,173)	0,752
Streptococcus sanguinis	0,17 (0,003-8,662)	0,378	5,13 (0,070-374,264)	0,455
Clostridium sensu stricto C. perfringens	1,10 (0,686-1,776)	0,685	2,34 (0,814-6,733)	0,114

Results controlled for confounder variables: gender, age at the time of surgery in years, BMI in kg/m2, diabetes and smoking status

Validity Testing of 16s rRNA Sequencing

A swab was taken intraoperatively from 53 patients for microbial determination. Of these, 39 patients (73.6%) were found to be positive. A total of 42 positive swab results from different anatomical regions were available. 37 swabs (88.1) came from the area of the bile ducts, including gallbladder and bile. Four swabs (9.5%) of inserted stents were also performed and one swab (2.4%) was taken from the area of the pancreatic duct. During the validity check of the DNA sequencing results, only 39 of the 42 swabs could be compared, as no corresponding samples were received for sequencing in three patients. 82.1% of the swabs (32 out of 39) were fully confirmed by DNA-based sequencing. In RNA-based sequencing, complete agreement was present in 81.0% of cases (34 out of 42). A total of 118 bacteria were detected. DNA sequencing recorded 107 of 114 evaluable bacteria (sensitivity 93.9%). Four bacteria could not be checked due to missing sequencing samples. RNA sequencing was able to detect 110 of the 118 bacteria, which corresponds to a sensitivity of 93.2%.

A total of 29 different species were identified by cultural breeding: 16 species (55.2%) could be assigned to the *Proteobacteria*, eleven species to the *Firmicutes (37.9%)* and two species (6.9%) to the

Bacteroidetes. If the taxa distribution is considered separately by anatomical region, the majority of the species detected in the bile ducts could be assigned to the *Proteobacteria* (n=15 of 27; 55.5%). The smears of the stents also revealed a majority of this phylum (n=8 out of 12; 66.7%). Bacterial species of the phylum *Firmicutes* took up 37.0% (n=10 of 27) of the species within the bile ducts and were represented by 33.3% (n=4 of 12) in the stent swabs. Species of Phylum *Bacteroidetes* were found exclusively in biliary tract swabs (n=2 of 27; 7.4%). A swab of the pancreatic duct could only detect the species *Escherichia coli* (Proteobacteria).
